# Negative and positive control ranges in the bacterial reverse mutation test: JEMS/BMS collaborative study

**DOI:** 10.1186/s41021-018-0096-1

**Published:** 2018-04-04

**Authors:** Masayuki Kato, Kei-ichi Sugiyama, Toshiro Fukushima, Yasuyoshi Miura, Takumi Awogi, Shigetomo Hikosaka, Kumiko Kawakami, Madoka Nakajima, Masato Nakamura, Hajime Sui, Kumiko Watanabe, Atsushi Hakura

**Affiliations:** 1 CMIC Bioresearch Center, CMIC Pharma Science Co., Ltd., Hokuto-shi, Yamanashi, 408-0044 Japan; 2 Division of Genetics and Mutagenesis, National Institute of Health Sciences, Setagaya-ku, Tokyo, 158-8501 Japan; 3 Scientfic Product Assessment Center, Japan Tobacco Inc., -shi, Yokohama, Kanagawa 227-8512 Japan; 4 Otsuka Pharmaceutical Co., Ltd., Tokushima, 771-0192 Japan; 5 Global Quality Management Center, CANON INC., Kawasaki-shi, Kanagawa 213-8512 Japan; 6 Hatano Research Institute, Food and Drug Safety Center, -shi, Hadano, Kanagawa 257-8523 Japan; 7 Former University of Shizuoka, 52-1, Yada, Suruga-ku, Shizuoka-city, Shizuoka 422-8526 Japan; 8 General Testing Research Institute, Japan Oilstuff Inspectors’ Corporation, 2-15, 1-chome, Mikagetsukamachi, Higashinada-ku, Kobe-shi, Hyogo 658-0044 Japan; 9 Drug Safety, Taisho Pharmaceutical co., Ltd., Saitama-shi, Saitama 331-9530 Japan; 10 Tsukuba Drug Safety, Eisai Co., Ltd., Tsukuba-shi, Ibaraki 300-2635 Japan

**Keywords:** Bacterial reverse mutation test, Validation study, Negative control range, Positive control range

## Abstract

A large-scale study was conducted by multiple laboratories affiliated with the Japanese Environmental Mutagen Society and the Bacterial Mutagenicity Study Group to investigate possible proficiency indicators for the bacterial reverse mutation test with a preincubation procedure. Approximately 30 laboratories generated negative and positive control count data and dose-response curves of the positive control articles for the bacterial reverse mutation test, with assays conducted annually from 2013 to 2016. Overall, the majority of the negative and positive control counts for *Salmonella* Typhimurium strains TA100, TA1535, TA98, and TA1537, and *Escherichia coli* strain WP2*uvrA*, with and without S9 mix, were within the range of the means ±2× standard deviation. The negative counts were normally distributed (strains TA100, TA98, and WP2*uvrA*) or followed Poisson distribution (strains TA1535 and TA1537), and the positive control counts for all strains were approximately normally distributed. In addition, the distribution of the negative and positive control counts was relatively constant over the 4 years. The number of revertant colonies increased in a dose-dependent linear or exponential fashion up to the recommended doses for the respective positive control articles in Japan. These data are valuable for determining the acceptance criteria and an estimation of the laboratory proficiency for the bacterial reverse mutation test.

**Electronic supplementary material:**

The online version of this article (10.1186/s41021-018-0096-1) contains supplementary material, which is available to authorized users.

## Background

The bacterial reverse mutation test, known as the Ames test, is often used to identify and characterize the mutagenicity of chemicals in basic research, and to examine the safety of industrial products prior to approval by regulatory agencies [1–6]. The structural alerts for mutagenicity derived from the results are also used by regulatory agencies to predict mutagenic impurities using in silico analysis according to the International Council for Harmonisation of Technical Requirements for Pharmaceuticals for Human Use M7 guideline [7]. The bacterial reverse mutation test is an integral component of genotoxicity tests performed as part of the regulatory requirements in accordance with the principles of Good Laboratory Practice, and therefore, it is important to use appropriate indicators for evaluating and demonstrating laboratory proficiency in the test. For this purpose, the negative and positive control ranges, along with the dose-response relationships of the positive control articles, are considered to be promising candidates.

To refine and maintain the data quality and experimental techniques used for the reverse mutation test, the Japanese Environmental Mutagen Society (JEMS)/Bacterial Mutagenicity Study Group (BMS) collaboratively conducted validation studies over two 4-year periods (2006–2009 and 2013–2016). In this paper, we report the results obtained from the four JEMS/BMS validation studies conducted annually during the period 2013–2016. The number of participating laboratories per year was 36 in 2013, 30 in 2014, 27 in 2015, and 26 in 2016. Data were obtained for the negative and positive control counts and the dose-response curves of the respective positive control articles using the five tester strains with and without S9 mix. The positive control articles (AF-2, 2-(2-furyl)-3-(5-nitro-2-furyl) acrylamide; SA, sodium azide; 9AA, 9-aminoacridine hydrochloride; and 2AA, 2-aminoanthracene) and bacterial strains (*Salmonella enterica* subsp*. enterica* serovar Typhimurium strains TA100, TA1535, TA98, and TA1537, and *Escherichia coli* strain WP2*uvrA*) used were those recommended in the Organisation for Economic Co-operation and Development (OECD) guideline for the testing of chemicals 471 [6].

## Materials and methods

### Chemicals and materials

An Ames Test Positive Control AM Multi-set (Lot number M0048, Wako Pure Chemical Industries, Ltd., Osaka, Japan) was used to provide the positive controls. The set comprised 2-(2-furyl)-3-(5-nitro-2-furyl) acrylamide (AF-2, purity of 99.7%), sodium azide (SA, purity of 100.0%), 9-aminoacridine hydrochloride (9AA, purity of 99.4%), and 2-aminoanthracene (2AA, purity of 96.7%). AF-2, 9AA, and 2AA were dissolved in dimethyl sulfoxide (DMSO, purity of 100%; Wako Pure Chemical Industries), while SA was dissolved in purified water.

S9 fraction, prepared from phenobarbital/5,6-benzoflavone-pretreated male Sprague-Dawley rat liver, was purchased from Oriental Yeast Co., Ltd. (Tokyo, Japan) or Kikkoman Biochemifa Co. (Chiba, Japan). Different lots of S9 fraction were used throughout the 4-year study. The S9 mix used in the assays consisted of 10% (*v*/v) S9 fraction (~ 1.0 mg protein/plate), 100 mM Na_2_HPO_4_/NaH_2_PO_4_, 8 mM MgCl_2_, 33 mM KCl, 4 mM NADP, 4 mM NAD, and 5 mM glucose-6-phosphate.

The *S.* Typhimurium top agar consisted of Bacto agar (0.6% (*w*/*v*) final concentration) and NaCl (0.5% (w/v) final concentration) dissolved in purified water supplemented with 0.05 mM L-histidine and 0.05 mM D-biotin. The *E. coli* top agar was the same as that used for *S.* Typhimurium, minus the D-biotin. Minimum glucose agar plates were obtained from Oriental Yeast Co. or Kyokuto Pharmaceutical Industrial Co. (Tokyo, Japan).

### Bacterial strains

The tester strains used were *S.* Typhimurium TA98 (*hisD3052*/*rfa*/*∆uvrB*/pKM101), TA100 (*hisG46*/*rfa*/*∆uvrB*/pKM101), TA1535 (*hisG46*/*rfa*/*∆uvrB*), and TA1537 (*hisC3076*/*rfa*/*∆uvrB*), and *E. coli* WP2*uvrA* (*trpE*/*uvrA*). These strains are recommended for use in the Ames test by Organisation for Economic Cooperation and Development (OECD) Guideline 471 [6], and this combination of strains is used in the majority of Japanese laboratories. Phenotypic characteristics of each of the strains, such as amino acid deficiencies (*his* for the *Salmonella* strains and *trp* for the *E. coli* strain), sensitivity to crystal violet (*rfa*), ampicillin resistance (pKM101), and sensitivity to ultraviolet light (*∆uvrB* for the *Salmonella* strains and *∆uvrA* for the *E. coli* strain), were confirmed as described previously [4, 5, 8, 9] prior to use in each laboratory. In addition, the frozen stock culture of each strain was also confirmed to have responses within each laboratory’s historical ranges to the negative and positive controls in advance of testing.

### Assay conditions

The reverse mutation test was conducted using a preincubation procedure according to the members’ own assay conditions, which had been established or confirmed to be valid through JEMS/BMS validation studies or seminars.

To obtain bacterial cells in early stationary phase, frozen stock cultures of each strain were inoculated into a conical flask or L-tube containing nutrient broth medium (2.5% (*w*/*v*); Oxoid nutrient broth No. 2, Oxoid Ltd., Hampshire, United Kingdom), and then either stored for > 3 h at approximately 4 °C prior to culture, or cultured immediately in a shaking incubator for between 7 and 10 h at 37 °C. Depending on the laboratory, the shaking incubators were set at various speeds (e.g. 140 rpm for a 100-mL conical flask, or 50 strokes/min for a 25-mL L-tube). The cell densities of each culture were confirmed to be > 1 × 10^9^ cells/mL by measuring optical density at 660 nm. For the assays carried out with S9 mix, 0.1 mL of the negative (vehicle) or positive control solution was added to a test tube, to which 0.5 mL of S9 mix and 0.1 mL of bacterial culture were added. For assays carried out in the absence of S9 mix, 100 mM sodium phosphate buffer (pH 7.4) was used in place of S9 mix. After mixing, the test tubes were preincubated for 20 min at 37 °C in a shaking water bath (between 70 and 150 strokes/min). Following preincubation, a 2-mL volume of pre-warmed (45 °C) top agar was added to each tube and mixed. Each mixture was then immediately poured onto the surface of minimal-glucose agar plates. After the top agar hardened, plates were incubated for 48 h at 37 °C. Each assay was conducted in duplicate (two plates per dose).

Water and DMSO were used as the negative (vehicle) controls for SA and for the other positive control articles, respectively. The positive control articles and doses used for each strain are listed in Additional file 1: Table S1. The maximum dose for each positive control was as recommended by the Japan Industrial Safety and Health Association [9]. The number of revertant colonies induced at the highest doses were used as the positive control counts for the assay.

### Data analysis

Mean and standard deviation (SD) were calculated from the experimental data generated by each laboratory using Excel (Microsoft, Redmond, WA, USA). The D’Agostino-Pearson and Kolmogorov-Smirnov tests were performed to evaluate the normality of data distribution, also using Excel [10]. A *p*-value < 0.05 was considered to be statistically significant.

## Results and discussion

### Negative (solvent) control data

**Fig. 1:**
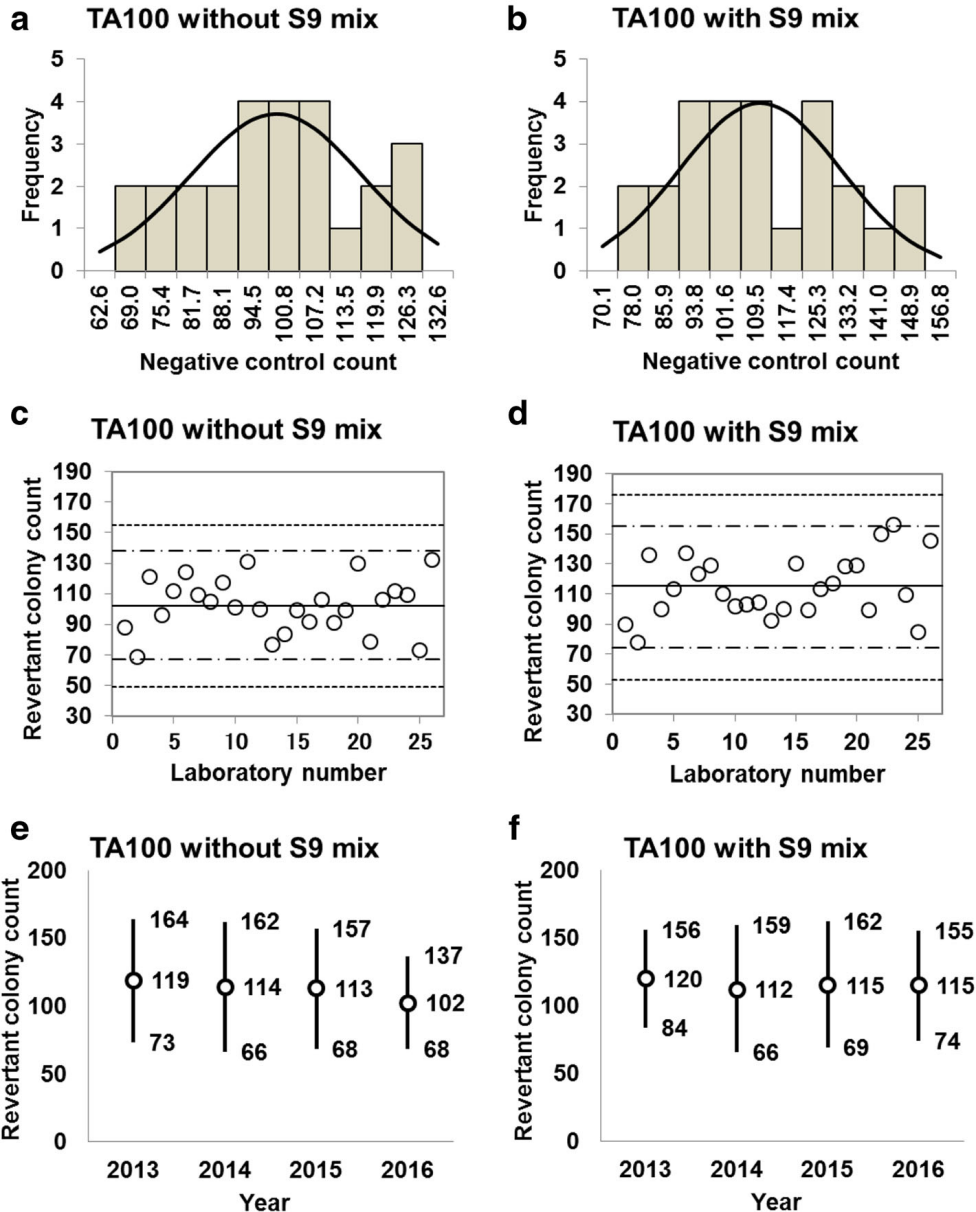
Negative control counts, and their subsequent analysis, for *Salmonella* Typhimurium strain TA100 with and without S9 mix. Histograms show the negative control counts, and the curves indicate the expected values calculated based on the assumption that the negative control counts follow a normal distribution, without (**a**) and with (**b**) S9 mix. Scatter plots showing the negative control counts generated by each participating laboratory without (**c**) and with (**d**) S9 mix are also shown, where the inner horizontal lines (−_˙_-) indicate the mean ± 2× standard deviation (SD), and outer horizontal lines (−--) indicate the mean ± 3× SD. The data shown in panels (**a**) to (**d**) are taken from the study conducted in 2016. The mean ± 2× SD values for the negative control counts for each individual year without (**e**) and with (**f**) S9 mix are also presented

**Fig. 2:**
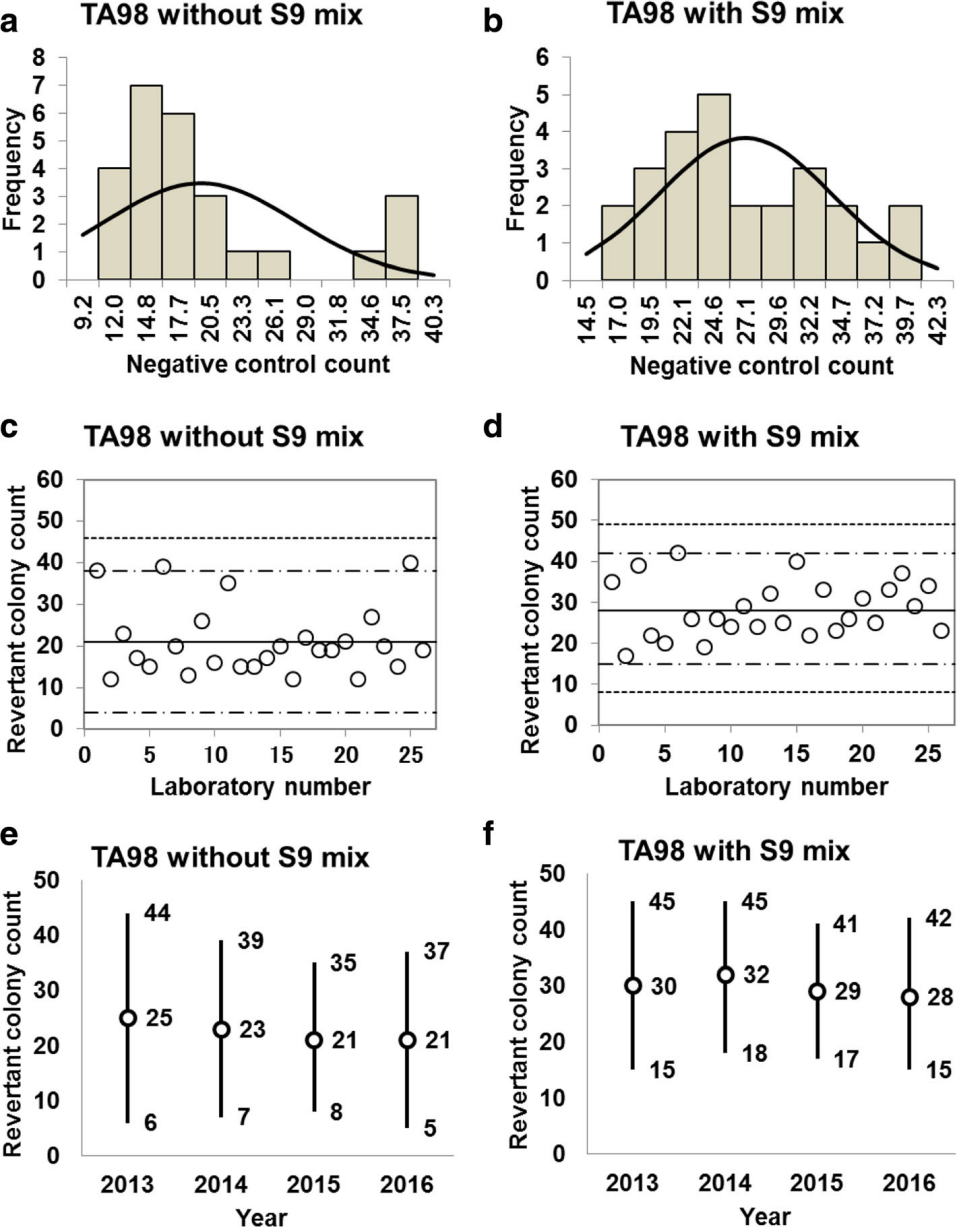
Negative control counts, and their subsequent analysis, for *Salmonella* Typhimurium strain TA98 with and without S9 mix. Scatter plots showing the negative control counts generated by each participating laboratory without (**c**) and with (**d**) S9 mix are also shown, where the inner horizontal lines (−_˙_-) indicate the mean ± 2× standard deviation (SD), and outer horizontal lines (−--) indicate the mean ± 3× SD. The data shown in panels (**a**) to (**d**) are taken from the study conducted in 2016. The mean ± 2× SD values for the negative control counts for each individual year without (**e**) and with (**f**) S9 mix are also presented

**Fig. 3:**
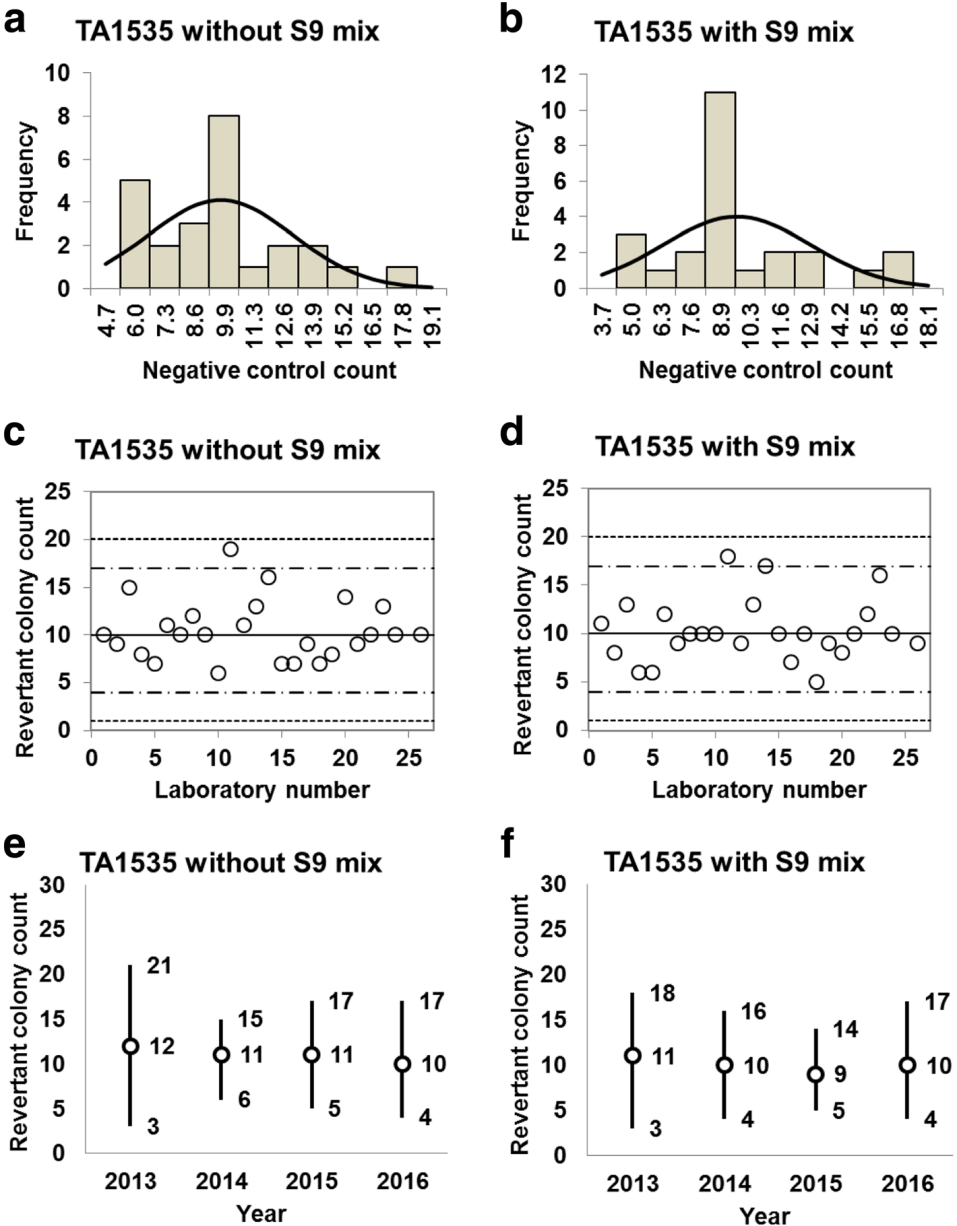
Negative control counts, and their subsequent analysis, for *Salmonella* Typhimurium strain TA1535 with and without S9 mix. Scatter plots showing the negative control counts generated by each participating laboratory without (**c**) and with (**d**) S9 mix are also shown, where the inner horizontal lines (−_˙_-) indicate the mean ± 2× standard deviation (SD), and outer horizontal lines (−--) indicate the mean ± 3× SD. The data shown in panels (**a**) to (**d**) are taken from the study conducted in 2016. The mean ± 2× SD values for the negative control counts for each individual year without (**e**) and with (**f**) S9 mix are also presented

**Fig. 4:**
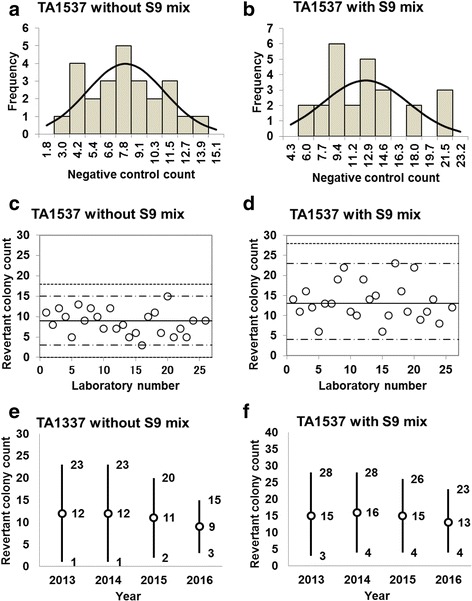
Negative control counts, and their subsequent analysis, for *Salmonella* Typhimurium strain TA1537 with and without S9 mix. Scatter plots showing the negative control counts generated by each participating laboratory without (**c**) and with (**d**) S9 mix are also shown, where the inner horizontal lines (−_˙_-) indicate the mean ± 2× standard deviation (SD), and outer horizontal lines (−--) indicate the mean ± 3× SD. The data shown in panels (**a**) to (**d**) are taken from the study conducted in 2016. The mean ± 2× SD values for the negative control counts for each individual year without (**e**) and with (**f**) S9 mix are also presented

**Fig. 5:**
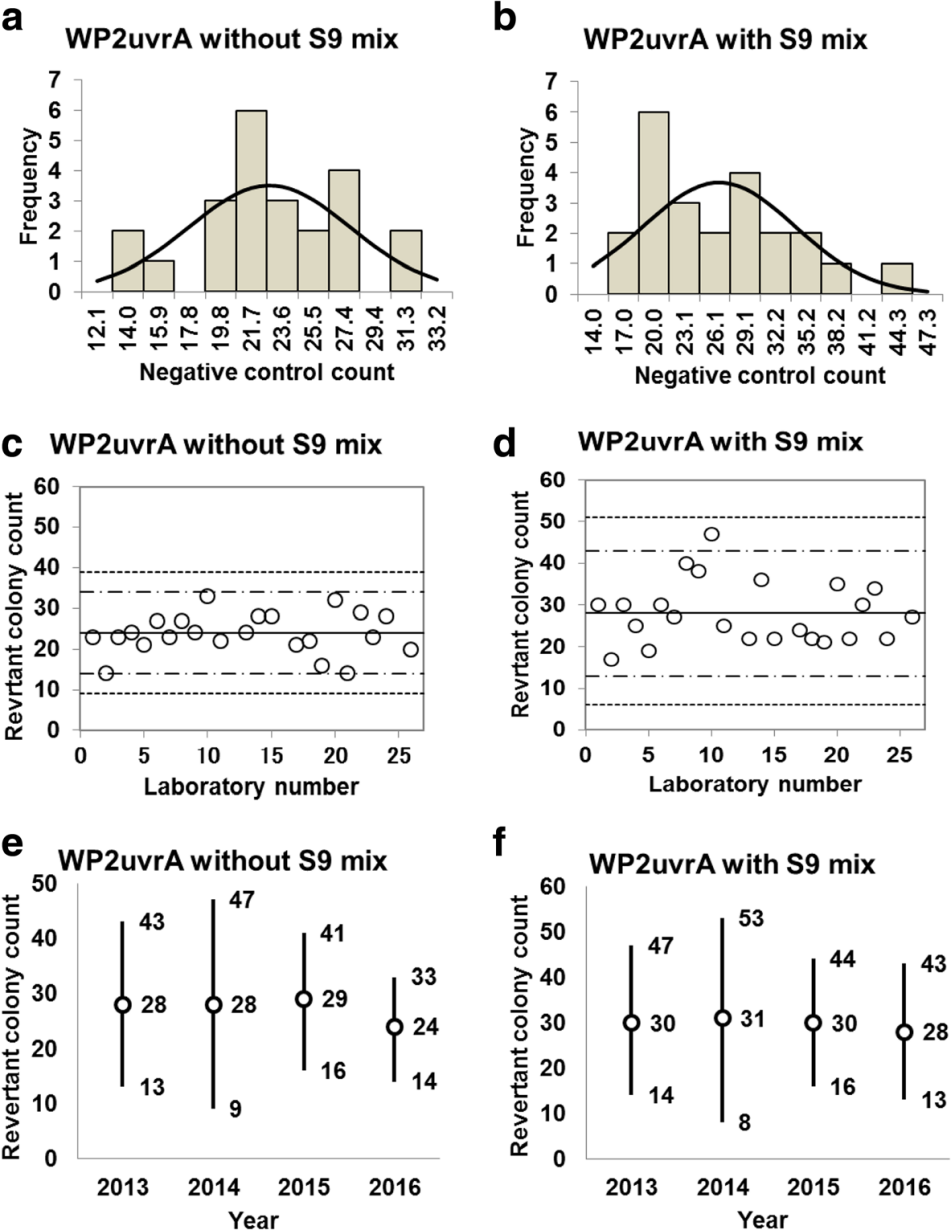
Negative control counts, and their subsequent analysis, for *Salmonella* Typhimurium strain WP2*uvrA* with and without S9 mix. Scatter plots showing the negative control counts generated by each participating laboratory without (**c**) and with (**d**) S9 mix are also shown, where the inner horizontal lines (−_˙_-) indicate the mean ± 2× standard deviation (SD), and outer horizontal lines (−--) indicate the mean ± 3× SD. The data shown in panels (**a**) to (**d**) are taken from the study conducted in 2016. The mean ± 2× SD values for the negative control counts for each individual year without (**e**) and with (**f**) S9 mix are also presented

Histograms, along with their corresponding estimated frequency curves generated under the assumption that the counts were normally distributed, were generated from the negative control counts (the mean number of revertant colonies/plate) in the absence and presence of S9 mix for strains TA100 (Fig. 1a and b), TA98 (Fig. 2a and b), TA1535 (Fig. 3a and b), TA1537 (Fig. 4a and b), and WP2*uvrA* (Fig. 5a and b). These data were provided by 23–26 participating laboratories in 2016.

D’Agostino-Pearson and Kolmogorov-Smirnov tests failed to reject the null hypothesis that the negative control counts were normally distributed for all five strains. In addition, the absolute values of the skewness and/or kurtosis for the five strains were less than 1.0, except for a value of 1.22 calculated for TA98 in the absence of S9 mix (Table 1). Most researchers tend to categorize absolute values of skewness and kurtosis of less than 1.0 as an indication of slight non-normality, values between 1.0 and about 2.3 as moderate non-normality, and values greater than 2.3 as severe non-normality. Therefore, the negative control counts of the strains with a relatively high number of negative control counts were deemed to be normally distributed (strains TA100, TA98, and WP2*uvrA*, both with and without S9 mix), as seen in Fig. 1a and b, Fig. 2a and b, and Fig. 5a and b. In contrast, the negative control counts of the strains with a relatively low number of negative control counts can be deemed to approximately follow Poisson distribution, as the mean values were almost equal to the variance (strains TA1535 and TA1537, both with and without S9 mix), as seen in Table 1.

**Table 1:** Statistics for negative control data obtained in this study

Statistics	TA100		TA98		TA1535		TA1537		WP2*uvrA*	
	-S9	+S9	-S9	+S9	-S9	+S9	-S9	+S9	-S9	+S9
No. of data	26	26	26	26	25	25	25	25	23	23
Mean	102	115	21	28	10	10	9	13	24	28
SD	17.34	20.06	8.23	6.66	3.10	3.17	2.95	4.60	4.87	7.33
Variance	312.57	418.66	70.36	46.06	10.01	10.48	9.08	22.01	24.75	56.23
Min	69	78	12	17	6	5	3	6	14	17
Max	132	156	40	42	19	18	15	23	33	47
Kurtosis	− 0.63	− 0.64	0.58	−0.73	0.88	0.64	− 0.63	− 0.24	0.04	0.29
Skewness	−0.09	0.30	1.22	0.40	0.98	0.80	0.15	0.54	−0.25	0.82
2SD-	68	74	5	15	4	4	3	4	14	13
2SD+	137	156	37	42	17	17	15	23	33	43
3SD-	50	54	−4	8	1	1	0	0	9	6
3SD+	154	175	46	48	20	20	17	27	38	50

*Min* minimum count, *max* maximum count, *2SD−* mean − 2× standard deviation, *2SD+* mean + 2× standard deviation, *3SD−* mean − 3× standard deviation, *3SD+* mean + 3× standard deviation

The negative control counts generated by each participating laboratory are shown in panels (c) and (d) of Figs. 1, 2, 3, 4, and 5. Almost all of the negative counts for each of the strains with and without S9 mix were within the range of the mean ± 2× SD, and counts from all laboratories were within the mean ± 3× SD, indicating that there were no outliers. As shown in panels (e) and (f) of Figs. 1, 2, 3, 4, and 5, there was little variance in the range of colony count values for each strain between each of the four years included in the study period. These findings indicate that laboratories using well-controlled assays carried out by proficient researchers can provide stable or consistent data. These negative control counts coincide with those reported previously [4, 5, 9, 11].

### Dose-response curves of positive control articles

**Fig. 6:**
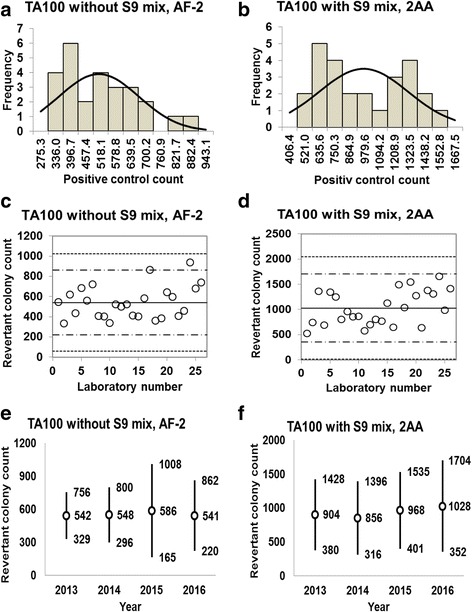
Positive control counts, and their subsequent analysis, for *Salmonella* Typhimurium strain TA100 with and without S9 mix. Histograms show the negative control counts, and the curves indicate the expected values calculated based on the assumption that the negative control counts follow a normal distribution, without (**a**) and with (**b**) S9 mix. Scatter plots showing the negative control counts generated by each participating laboratory without (**c**) and with (**d**) S9 mix are also shown, where the inner horizontal lines (−_˙_-) indicate the mean ± 2× standard deviation (SD), and outer horizontal lines (−--) indicate the mean ± 3× SD. The data shown in panels (**a**) to (**d**) are taken from the study conducted in 2016. The mean ± 2× SD values for the negative control counts for each individual year without (**e**) and with (**f**) S9 mix are also presented. The doses used were 0.01 μg/plate for AF-2 in the absence of S9 mix, and 1.0 μg/plate for 2AA in the presence of S9 mix

**Fig. 7:**
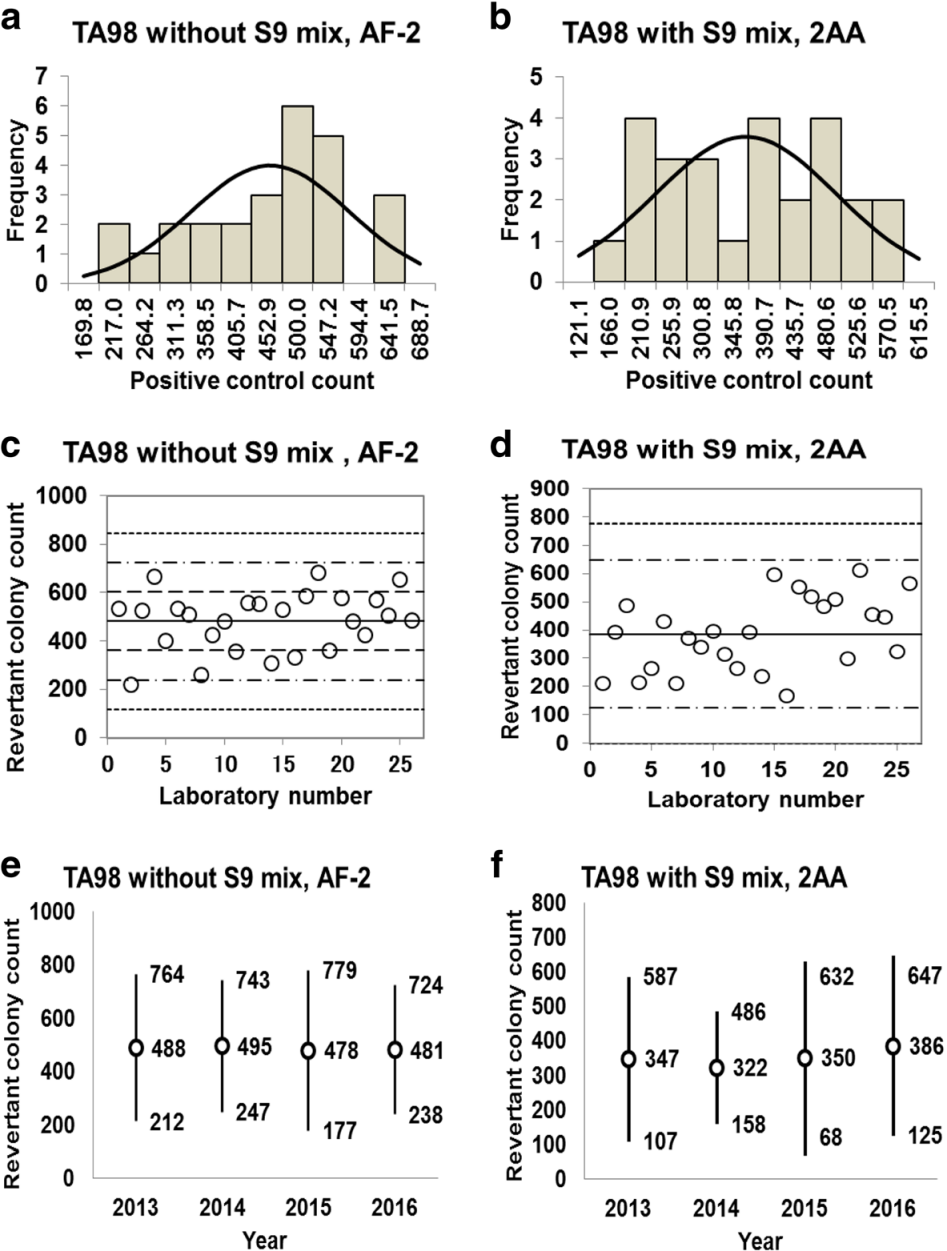
Positive control counts, and their subsequent analysis, for *Salmonella* Typhimurium strain TA98 with and without S9 mix. Histograms show the negative control counts, and the curves indicate the expected values calculated based on the assumption that the negative control counts follow a normal distribution, without (**a**) and with (**b**) S9 mix. Scatter plots showing the negative control counts generated by each participating laboratory without (**c**) and with (**d**) S9 mix are also shown, where the inner horizontal lines (−_˙_-) indicate the mean ± 2× standard deviation (SD), and outer horizontal lines (−--) indicate the mean ± 3× SD. The data shown in panels (**a**) to (**d**) are taken from the study conducted in 2016. The doses used were 0.1 μg/plate for AF-2 in the absence of S9 mix, and 0.5 μg/plate for 2AA in the presence of S9 mix

**Fig. 8:**
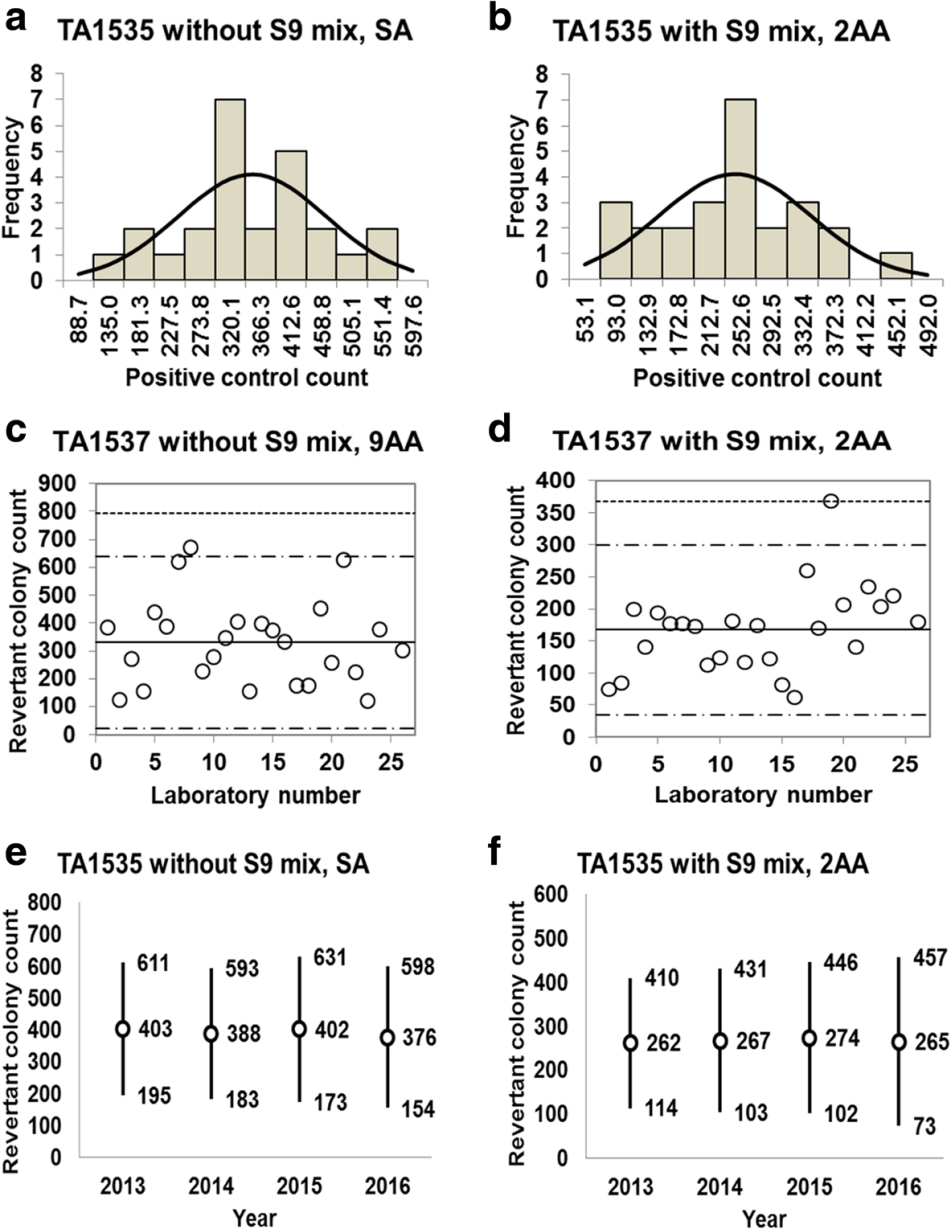
Positive control counts, and their subsequent analysis, for *Salmonella* Typhimurium strain TA1535 with and without S9 mix. Histograms show the negative control counts, and the curves indicate the expected values calculated based on the assumption that the negative control counts follow a normal distribution, without (**a**) and with (**b**) S9 mix. Scatter plots showing the negative control counts generated by each participating laboratory without (**c**) and with (**d**) S9 mix are also shown, where the inner horizontal lines (−_˙_-) indicate the mean ± 2× standard deviation (SD), and outer horizontal lines (−--) indicate the mean ± 3× SD. The data shown in panels (**a**) to (**d**) are taken from the study conducted in 2016. The doses used were 0.5 μg/plate for SA in the absence of S9 mix, and 2.0 μg/plate for 2AA in the presence of S9 mix

**Fig. 9:**
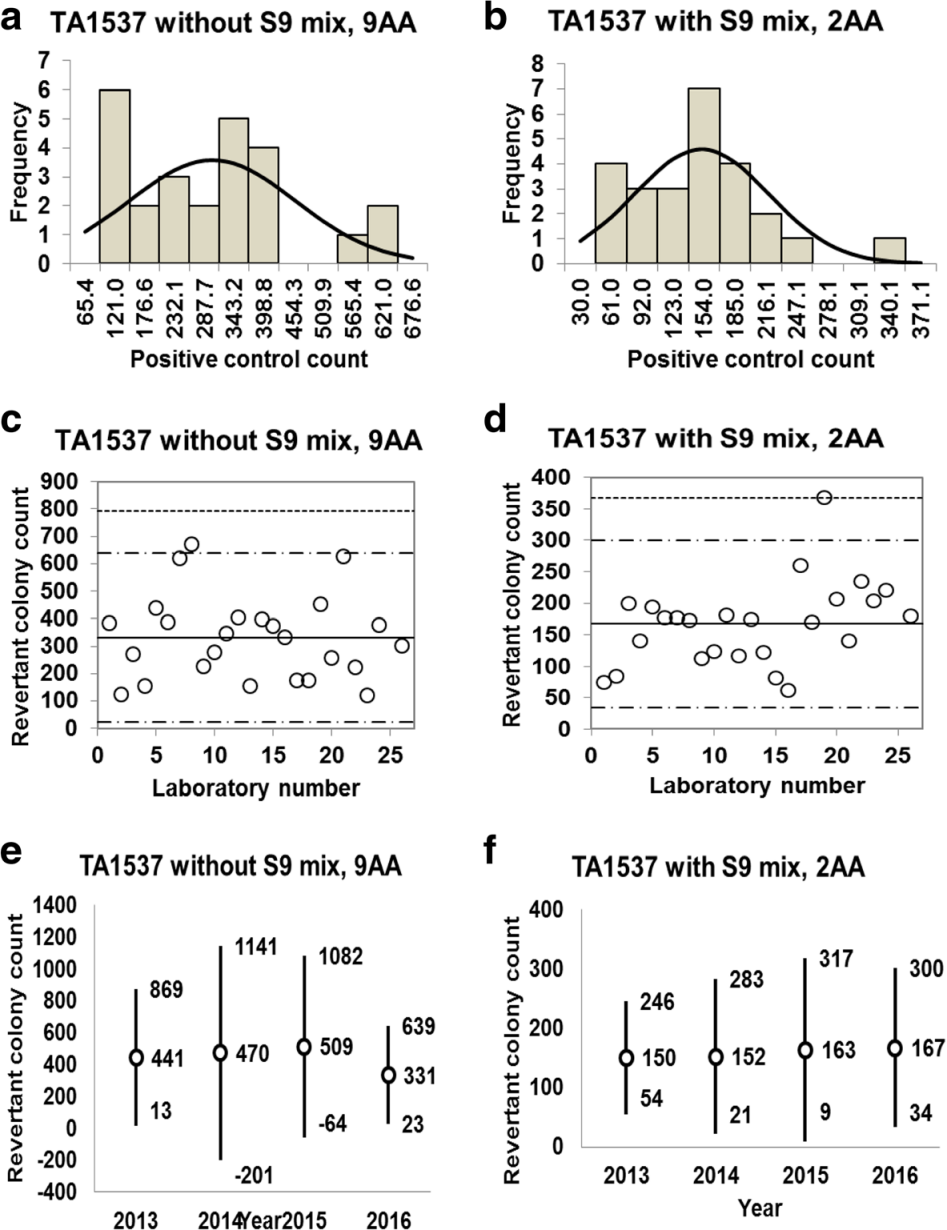
Positive control counts, and their subsequent analysis, for *Salmonella* Typhimurium strain TA1537 with and without S9 mix. Histograms show the negative control counts, and the curves indicate the expected values calculated based on the assumption that the negative control counts follow a normal distribution, without (**a**) and with (**b**) S9 mix. Scatter plots showing the negative control counts generated by each participating laboratory without (**c**) and with (**d**) S9 mix are also shown, where the inner horizontal lines (−_˙_-) indicate the mean ± 2× standard deviation (SD), and outer horizontal lines (−--) indicate the mean ± 3× SD. The data shown in panels (**a**) to (**d**) are taken from the study conducted in 2016. The doses used were 80 μg/plate for 9AA in the absence of S9 mix, and 2.0 μg/plate for 2AA in the presence of S9 mix

**Fig. 10:**
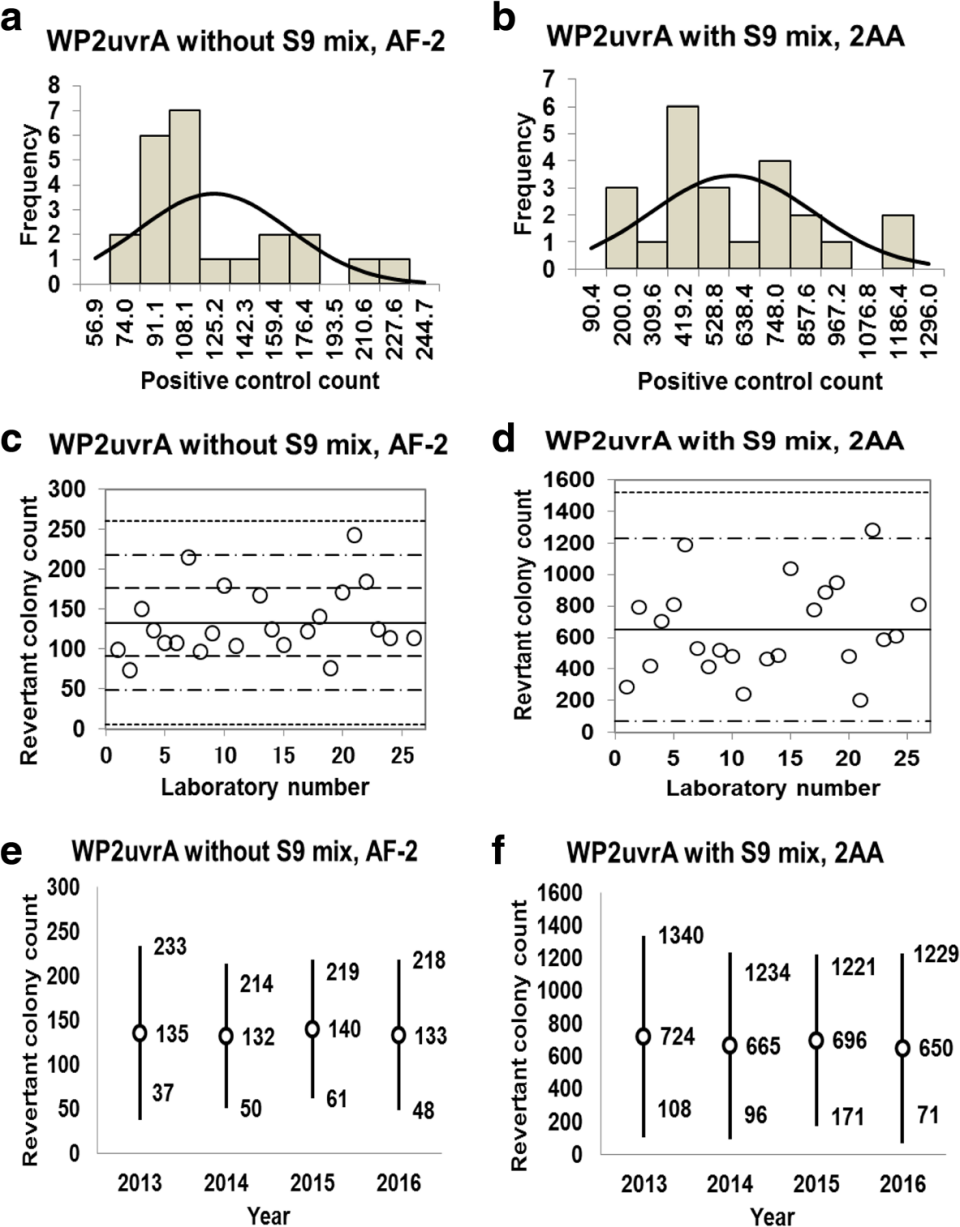
Positive control counts, and their subsequent analysis, for *Escherichia coli* strain WP2*uvrA* with and without S9 mix. Histograms show the negative control counts, and the curves indicate the expected values calculated based on the assumption that the negative control counts follow a normal distribution, without (**a**) and with (**b**) S9 mix. Scatter plots showing the negative control counts generated by each participating laboratory without (**c**) and with (**d**) S9 mix are also shown, where the inner horizontal lines (−_˙_-) indicate the mean ± 2× standard deviation (SD), and outer horizontal lines (−--) indicate the mean ± 3× SD. The data shown in panels (**a**) to (**d**) are taken from the study conducted in 2016. The doses used were 0.01 μg/plate for SA in the absence of S9 mix, and 10 μg/plate for 2AA in the presence of S9 mix

The dose-response curves for the five strains at three different doses of each control article (D1, D2, and D3; Additional file 1: Table S1) in the presence or absence of S9 mix are shown in Figs. 6, 7, 8, 9, and 10. The data for the dose-response curves were generated by 24–27 JEMS/BMS laboratories who participated in the validation study in 2016. All of the maximum doses designated “D3” (Additional file 1: Table S1) are those recommended for each positive control article by the Japan Industrial Safety and Health Association [9], and are in-line with doses frequently used in many Japanese laboratories. Linear relationships between the dose and the number of revertant colonies were observed for strains TA100, TA98, and TA1535, both with and without S9 mix (Additional file 2: Figure S1, Additional file 3: Figure S2, and Additional file 4: Figure S3), while exponential relationships between the dose and number of revertant colonies were observed for strains TA1537 and WP2*uvrA*, with and without S9 mix (Additional file 5: Figure S4 and Additional file 6: Figure S5).

Two laboratories showed a reduced number of revertant colonies at the maximum positive control article dose in the absence of S9 mix: strain TA98 treated with AF-2 (Additional file 3: Figure S2), and strain TA1537 treated with 9AA (Additional file 5: Figure S4). The reduced activity seen at the highest dose of AF-2 is an example of why it may be best to use positive control doses that are not at or near the top of the dose-response curves, but on the ascending portion of the curves. If the response falls within the flat portion at the top of the curve, then changes in the potency of the response may not be noticeable. Thus, this study provides corroborating evidence that the recommended positive doses outlined by the Japan Industrial Safety and Health Association [9] are appropriate. The mutagenic and cytotoxic potential of AF-2 is reported to vary depending on the pre-culture conditions. For example, this compound showed decreased mutagenicity but increased cytotoxicity towards bacteria cultured under anaerobic conditions compared with those cultured under aerobic conditions [11]. Therefore, the aberrant results obtained in the current study possibly indicate inappropriate pre-culture conditions. In the case of 9AA, we suspect some sort of technical error may account for the reduced number of revertant colonies obtained at the maximum dose by one laboratory.

### Positive control data

Histograms and their corresponding estimated frequency curves (generated under the assumption that the counts were normally distributed) were produced from the positive control counts (the mean number of revertant colonies/plate) in the absence and presence of S9 mix for strains TA100 (Fig. 6a and b), TA98 (Fig. 7a and b), TA1535 (Fig. 8a and b), TA1537 (Fig. 9a and b), and WP2*uvrA* (Fig. 10a and b)*.* These data were provided by 24–27 participating laboratories in 2016.

D’Agostino-Pearson and Kolmogorov-Smirnov tests failed to reject the null hypothesis that the positive control counts were normally distributed for all five strains under all test conditions, except for strain TA1537 treated with 2AA in the presence of S9 mix. The absolute values of the skewness and kurtosis were less than 1.0 in most cases, although the observed values were slightly greater than 1.0 for TA100 and TA98 treated with 2AA in the presence of S9 mix, and WP2*uvrA* treated with AF-2 in the absence of S9 mix (Table 2). Although the kurtosis value was 2.17 for TA1537 treated with 2AA in the presence of S9 mix, it was less than the cut-off value of 2.3, values above which are indicative of severe non-normality (Table 2). None of the positive control counts showed Poisson distribution because the variances were much larger than the mean values. Therefore, we concluded that all positive control counts for all strains were approximately normally distributed.

**Table 2:** Statistics for positive control data obtained in this study

Statistics	TA100		TA98		TA1535		TA1537		WP2*uvrA*	
	-S9	+S9	-S9	+S9	-S9	+S9	-S9	+S9	-S9	+S9
Positive control article and dose (μg/plate)	AF-2 (0.01)	2AA (1.0)	AF-2 (0.1)	2AA (0.5)	SA (0.5)	2AA (2.0)	9AA (80)	2AA (2.0)	AF-2 (0.01)	2AA (10)
No. of data	27	27	27	27	26	26	26	26	24	24
Mean	541	1028	481	386	376	265	331	167	133	650
SD	157.23	331.4	119.21	128.07	108.98	93.99	150.7	65.38	41.60	282.98
Min	336	521	217	166	135	93	121	61	74	200
Max	937	1656	684	611	593	488	671	368	243	1285
Kurtosis	0.13	−1.28	−0.30	−1.09	−0.03	−0.01	0.00	2.17	0.80	−0.23
Skewness	0.80	0.25	−0.45	0.05	−0.21	0.15	0.67	0.89	1.05	0.55
2SD-	226	365	243	130	158	77	29	36	50	84
2SD+	855	1691	719	643	594	453	632	298	216	1216
3SD-	69	34	123	2	49	−17	− 122	−29	8	−199
3SD+	1013	2022	839	771	703	547	783	363	258	1499

*Min* minimum count, *max* maximum count, *2SD−* mean − 2× standard deviation, *2SD+* mean + 2× standard deviation, *3SD−* mean − 3× standard deviation, *3SD+* mean + 3× standard deviation, *AF-2* 2-(2-furyl)-3-(5-nitro-2-furyl) acrylamide, *2AA* 2-aminoanthracene, *SA* sodium azide, *9AA* 9-aminoacridine hydrochloride

The positive control counts generated by each participating laboratory are shown in panels (c) and (d) of Figs. 6, 7, 8, 9, and 10. Almost all of the positive control counts were within the range of the mean ± 2× SD, and all counts were within the mean ± 3× SD, indicating that there were no outliers. As shown in panels (e) and (f) of Figs. 6, 7, 8, 9 and 10, there was little variance in the range of colony count values for each strain between each of the four years included in the study period. As with the negative control counts, these findings indicate that laboratories with well-controlled assays and highly proficient staff can provide stable or consistent data.

### Influence of different S9 manufactures/production lots on negative/positive control values and dose-response curves

S9 fraction prepared from the livers of male Sprague-Drawly rats pretreated with phenobarbital and 5,6-benzoflavon was used by all participating laboratories in 2016. The S9 fractions were purchased from two manufacturers. Ten laboratories used S9 fraction manufactured by Kikkoman, while 16 laboratories used S9 fraction manufactured by Oriental Yeast. However, as shown in Additional file 7: Figure S6, no large differences were observed in the negative/positive control values or dose-response curves between the S9 manufactures or among production lots.

### Relationships between negative and positive control counts

Scatter diagrams were generated to show the relationship between negative and positive control counts for each of the five strains with and without S9 mix (Additional file 8: Figure S7). The resulting low correlation coefficients suggested that there was no relationship between the negative and positive control counts.

## Conclusion

The data presented here, collected from a series of validation studies conducted collaboratively by proficient JEMS/BMS members, will be of use in determining possible acceptance criteria to confirm or demonstrate laboratory proficiency in the reverse mutation test.

## Additional files

Additional file 1: **Table S1.** Positive control articles and their doses used in this study (DOCX 18 kb)

Additional file 2: **Figure S1.** Dose-response curves of revertant *Salmonella* Typhimurium strain TA100 colonies following treatment with AF-2 in the absence of S9 mix (a), or with 2AA in the presence of S9 mix (b). Individual dose-response curves were generated using results produced by each participating laboratory in 2016 (different colors indicate different laboratories). The doses tested were 0.0025, 0.005, and 0.01 μg/plate for AF-2, and 0.25, 0.5, and 1.0 μg/plate for 2AA. (ODP 423 kb)

Additional file 3: **Figure S2.** Dose-response curves of revertant *Salmonella* Typhimurium strain TA98 colonies following treatment with AF-2 in the absence of S9 mix (a), or treatment with 2AA in the presence of S9 mix (b). Individual dose-response curves were generated using results produced by each participating laboratory in 2016 (different colors indicate different laboratories). The doses tested were 0.025, 0.05, and 0.1 μg/plate for AF-2, and 0.125, 0.25, and 0.5 μg/plate for 2AA. (ODP 434 kb)

Additional file 4: **Figure S3.** Dose-response curves of revertant *Salmonella* Typhimurium strain TA1535 colonies following treatment with SA in the absence of S9 mix (a), or with 2AA in the presence of S9 mix (b). Individual dose-response curves were generated using results produced by each participating laboratory in 2016 (different colors indicate different laboratories). The doses tested were 0.125, 0.25, and 0.5 μg/plate for SA, and 0.5, 1.0, and 2.0 μg/plate for 2AA. (ODP 411 kb)

Additional file 5: **Figure S4.** Dose-response curves of revertant *Salmonella* Typhimurium strain TA1537 colonies following treatment with 9AA in the absence of S9 mix (a), or with 2AA in the presence of S9 mix (b). Individual dose-response curves were generated using results produced by each participating laboratory in 2016 (different colors indicate different laboratories). The doses tested were 20, 40, and 80 μg/plate for 9AA, and 0.5, 1.0, and 2.0 μg/plate for 2AA. (ODP 343 kb)

Additional file 6: **Figure S5.** Dose-response curves of revertant *Escherichia coli* strain WP2*uvrA* colonies following treatment with AF-2 in the absence of S9 mix (a), or with 2AA in the presence of S9 mix (b). Individual dose-response curves were generated using results produced by each participating laboratory in 2016 (different colors indicate different laboratories). The doses tested were 0.0025, 0.005, and 0.01 μg/plate for AF-2, and 2.5, 5.0, and 10 μg/plate for 2AA. (ODP 342 kb)

Additional file 7: **Figure S6.** Individual dose-response curves showing the positive control articles in the presence of S9 mix were generated from data obtained by each participating laboratory (each laboratory is indicated by a different color). The doses (in μg/plate) for each positive control article are the same as those shown in Figs. 6, 7, 8, 9, and 10. S9 fraction was purchased from Kikkoman or Oriental Yeast, and while some laboratories used that same lots, other laboratories used different lots from the same manufacturers. Laboratory identification numbers are indicated on the right-hand side of each figure. (ODP 516 kb)

Additional file 8: **Figure S7.** Comparison of the negative and positive control counts for each strain with or without S9 mix. The doses shown in Figs. 6, 7, 8, 9, and 10 were used for comparison. The R^2^ values indicate the correlation coefficients of the linear regression lines. (ODP 62 kb)

## Abbreviations

2AA: 2-aminoanthracene
9AA: 9-aminoacridine hydrochloride
AF-2: 2-(2-furyl)-3-(5-nitro-2-furyl) acrylamide
BMS: Bacterial Mutagenicity Study Group
DMSO: Dimethyl sulfoxide
GLP: Good Laboratory Practice
JEMS: Japanese Environmental Mutagen Society
OECD: Organisation for Economic Cooperation and Development
SA: Sodium azide
SD: Standard deviation
