# Supplementary material for: Negative and positive control ranges in the bacterial reverse mutation test: JEMS/BMS collaborative study
Source: Genes Environ. 2018 Apr 4;40:7. doi: 10.1186/s41021-018-0096-1 (PMC5883876; doi:10.1186/s41021-018-0096-1)
Supplement: Supplementary file 1 — Table S1. Positive control articles and their doses used in this study (DOCX 18 kb) [file 41021_2018_96_MOESM1_ESM.docx]

**Table S1** Positive control articles and their doses used in this study

|  |  |  | Dose (µg/plate) | | |
| --- | --- | --- | --- | --- | --- |
| Strain | **S9** | **Compound** | **D1** | **D2** | **D3** |
| TA100 | − | AF-2 | 0.0025 | 0.005 | 0.01 |
|  | + | 2AA | 0.25 | 0.5 | 1.0 |
| TA98 | − | AF-2 | 0.025 | 0.05 | 0.1 |
|  | + | 2AA | 0.125 | 0.25 | 0.5 |
| TA1535 | − | SA | 0.125 | 0.25 | 0.5 |
|  | + | 2AA | 0.5 | 1.0 | 2.0 |
| TA1537 | − | 9AA | 20 | 40 | 80 |
|  | + | 2AA | 0.5 | 1.0 | 2.0 |
| WP2*uvrA* | − | AF-2 | 0.0025 | 0.005 | 0.01 |
|  | + | 2AA | 2.5 | 5.0 | 10 |

AF-2, 2-(2-furyl)-3-(5-nitro-2-furyl) acrylamide; 2AA, 2-aminoanthracene; SA, sodium azide; 9AA, 9-aminoacridine hydrochloride
