# Supplementary figures and images for: Negative and positive control ranges in the bacterial reverse mutation test: JEMS/BMS collaborative study
Source: Genes Environ. 2018 Apr 4;40:7. doi: 10.1186/s41021-018-0096-1 (PMC5883876; doi:10.1186/s41021-018-0096-1)

## Slide: page1
Figure S1
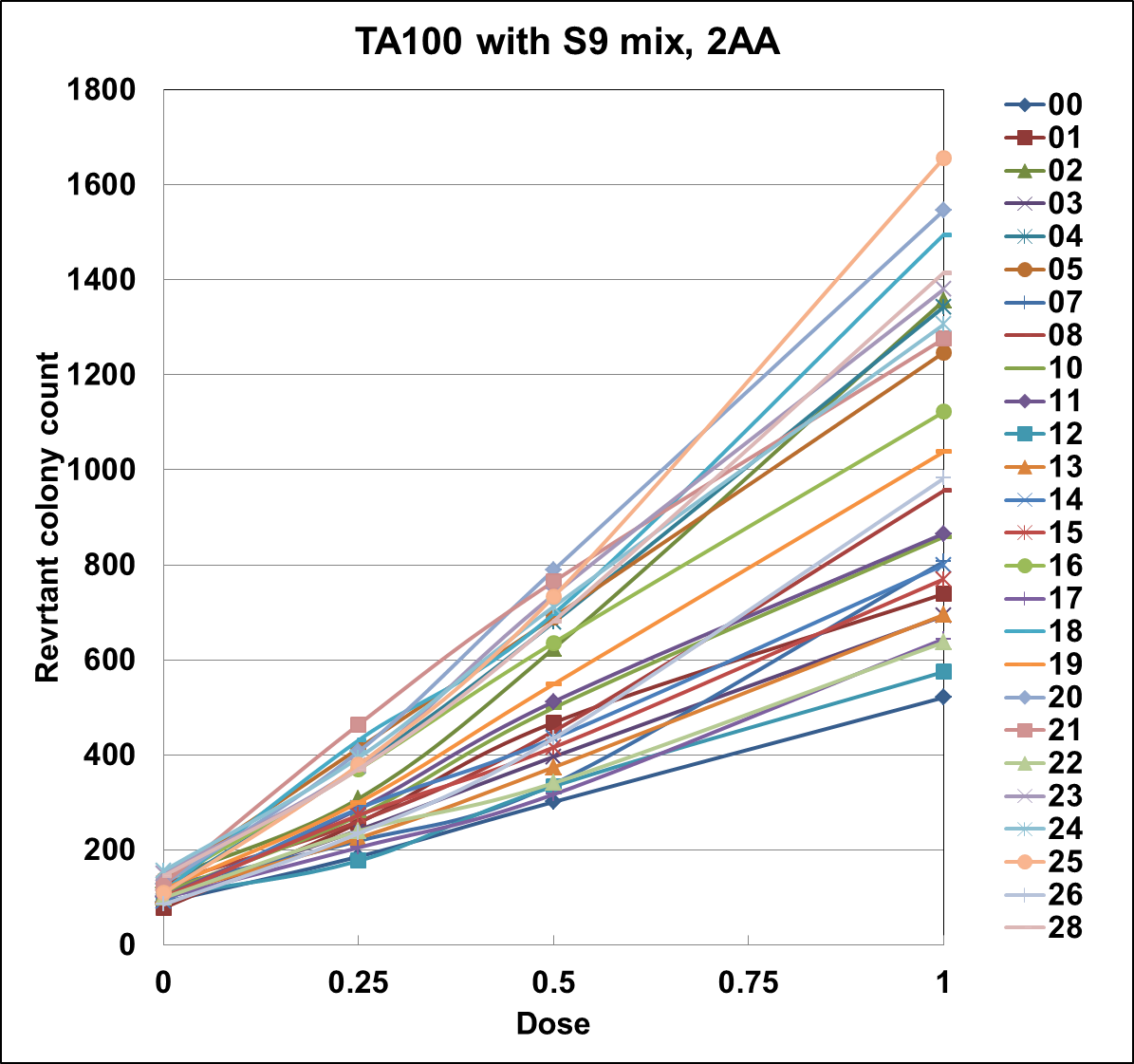

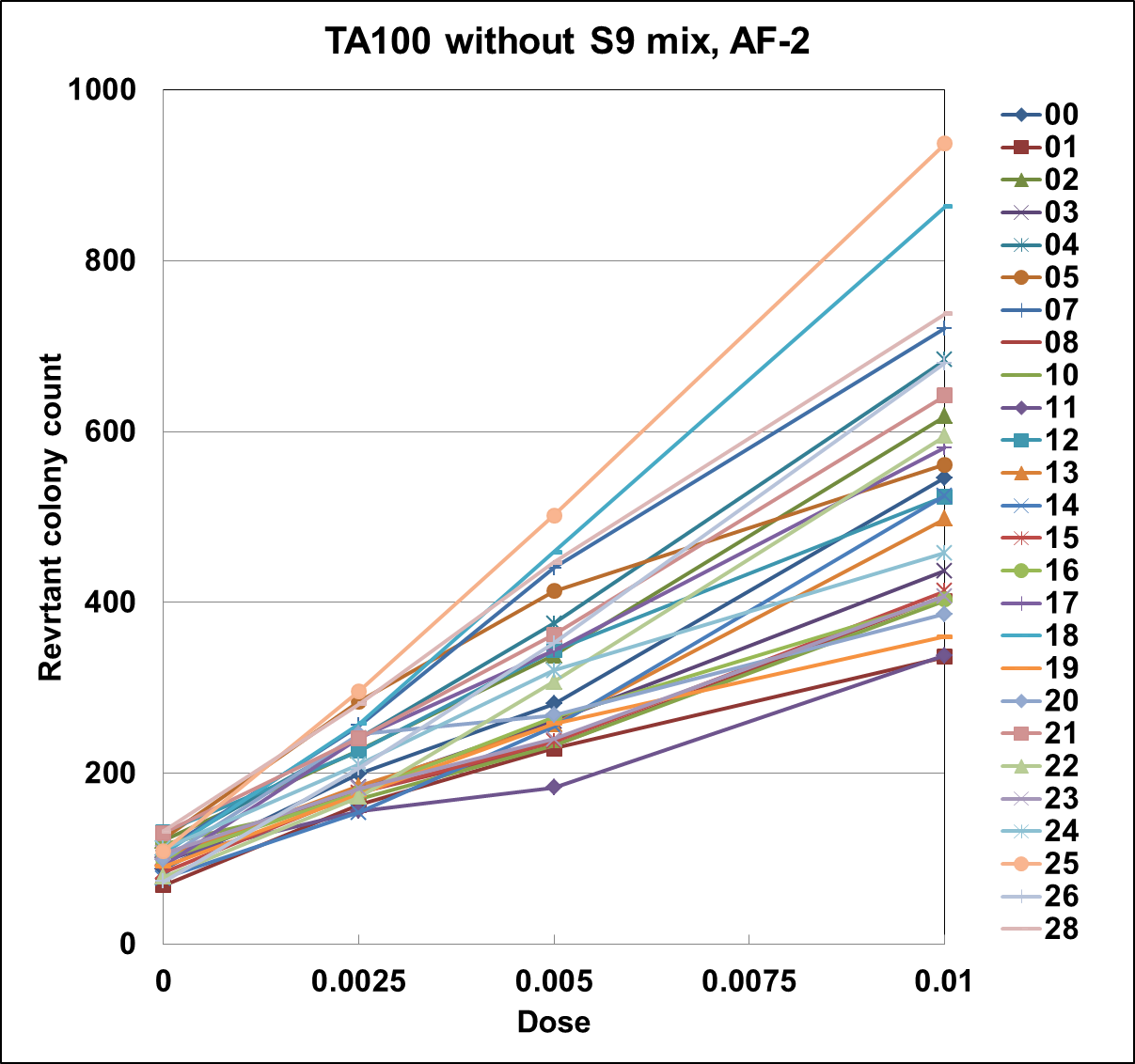

Supplement: Supplementary file 2 — Figure S1. Dose-response curves of revertant Salmonella Typhimurium strain TA100 colonies following treatment with AF-2 in the absence of S9 mix (a), or with 2AA in the presence of S9 mix (b). Individual dose-response curves were generated using results produced by each participating laboratory in 2016 (different colors indicate different laboratories). The doses tested were 0.0025, 0.005, and 0.01 μg/plate for AF-2, and 0.25, 0.5, and 1.0 μg/plate for 2AA. (ODP 423 kb) [file 41021_2018_96_MOESM2_ESM.odp]

## Slide: page1
Figure S2
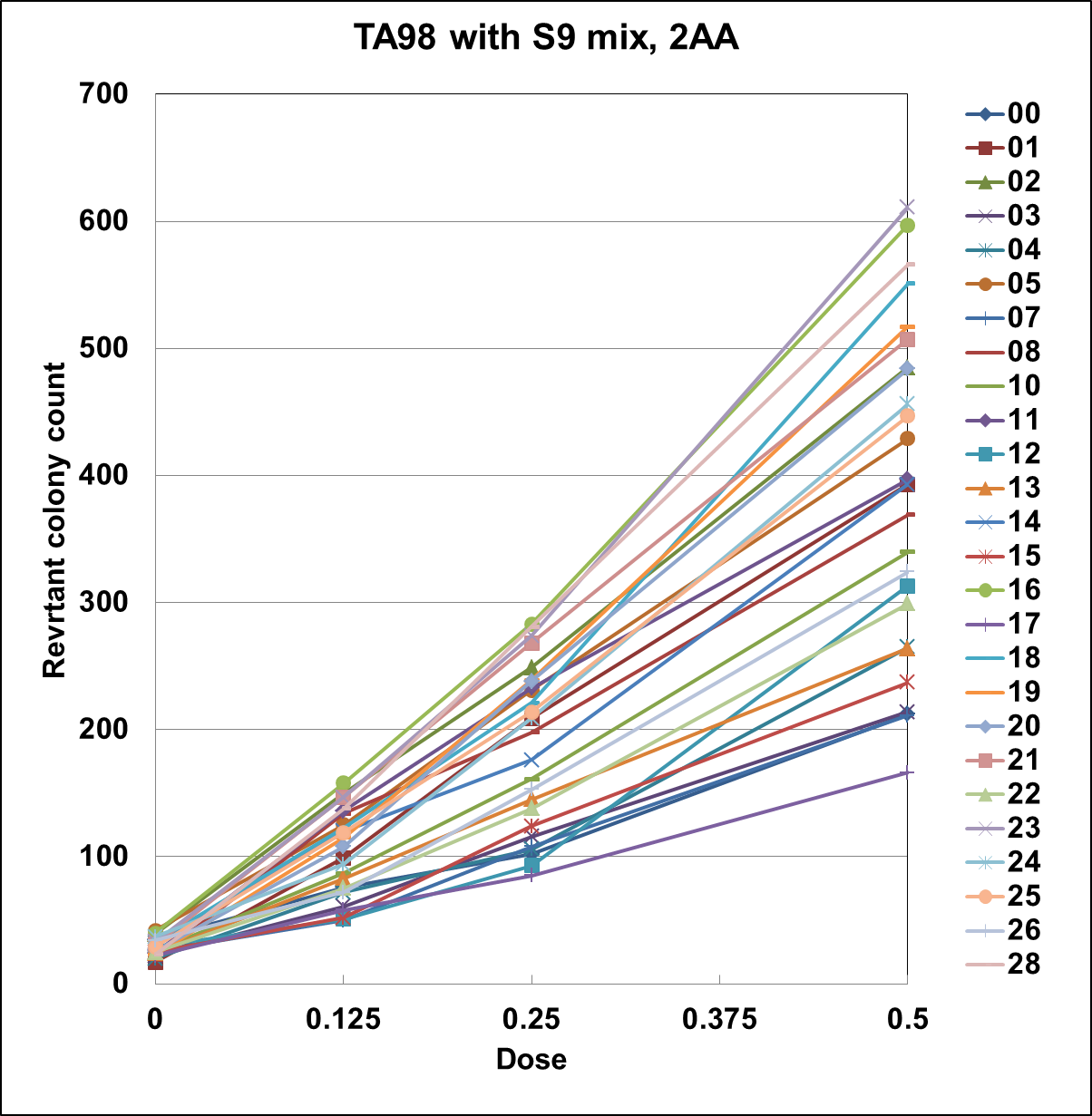

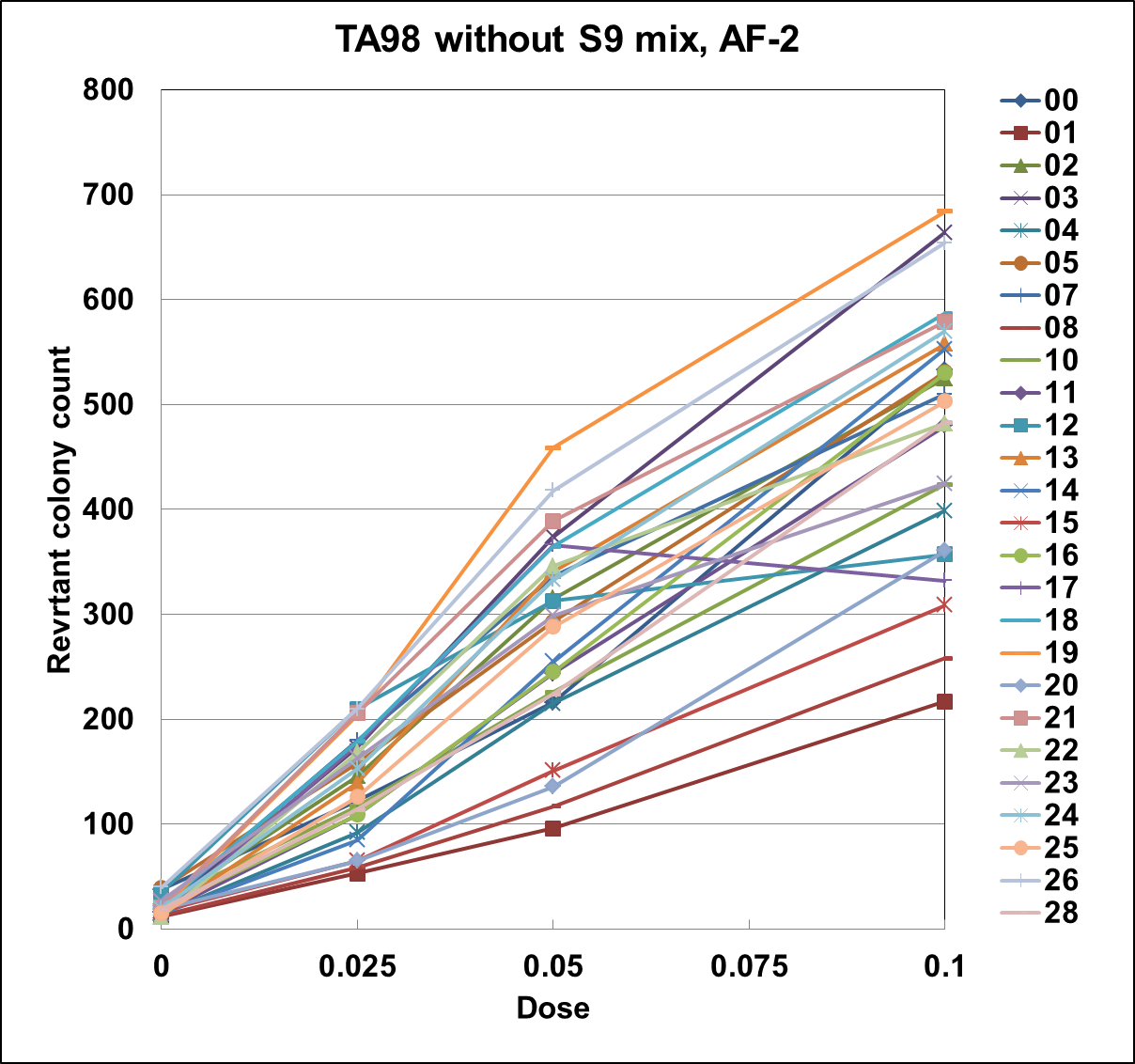

Supplement: Supplementary file 3 — Figure S2. Dose-response curves of revertant Salmonella Typhimurium strain TA98 colonies following treatment with AF-2 in the absence of S9 mix (a), or treatment with 2AA in the presence of S9 mix (b). Individual dose-response curves were generated using results produced by each participating laboratory in 2016 (different colors indicate different laboratories). The doses tested were 0.025, 0.05, and 0.1 μg/plate for AF-2, and 0.125, 0.25, and 0.5 μg/plate for 2AA. (ODP 434 kb) [file 41021_2018_96_MOESM3_ESM.odp]

## Slide: page1
Figure  S3
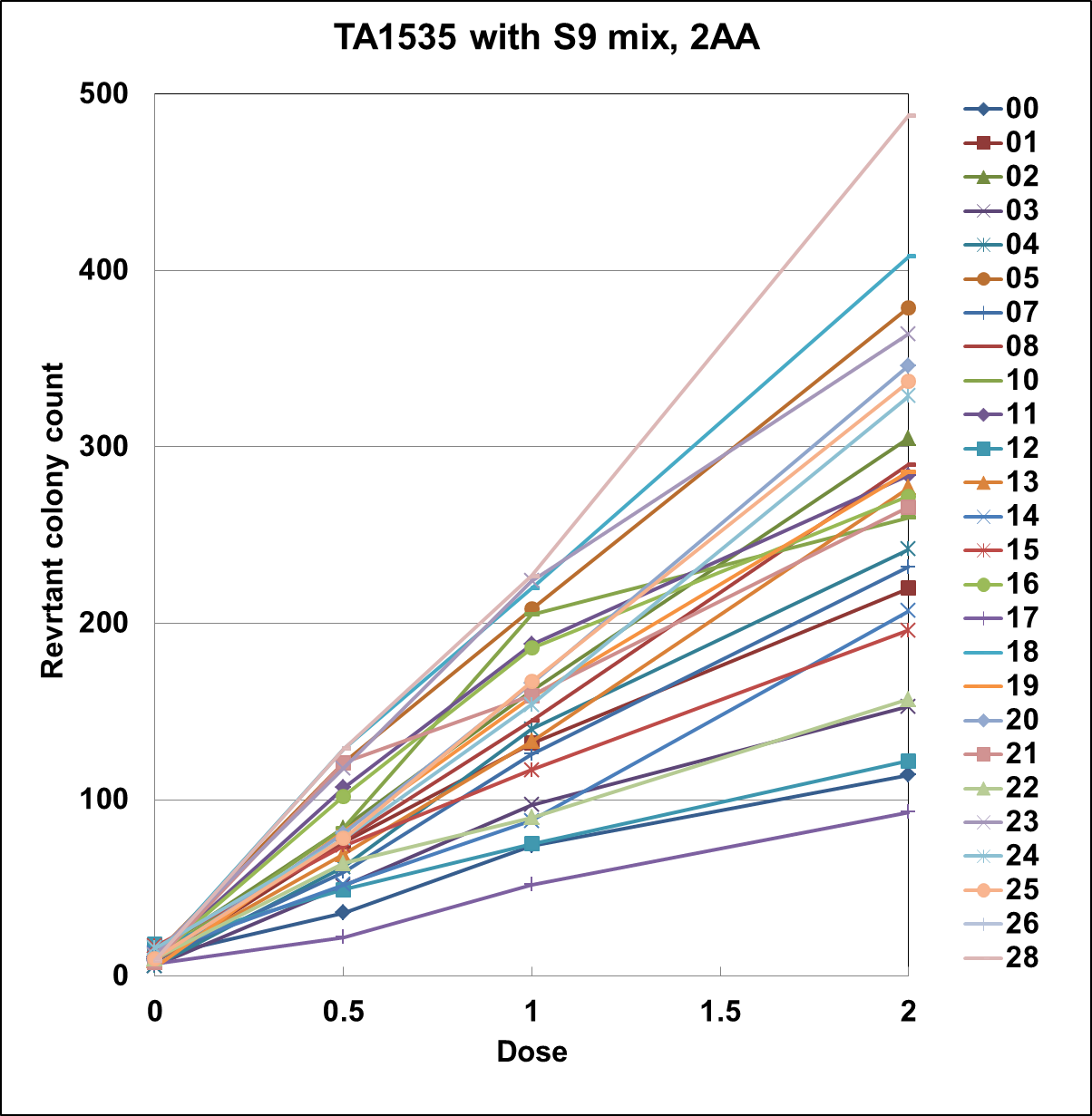

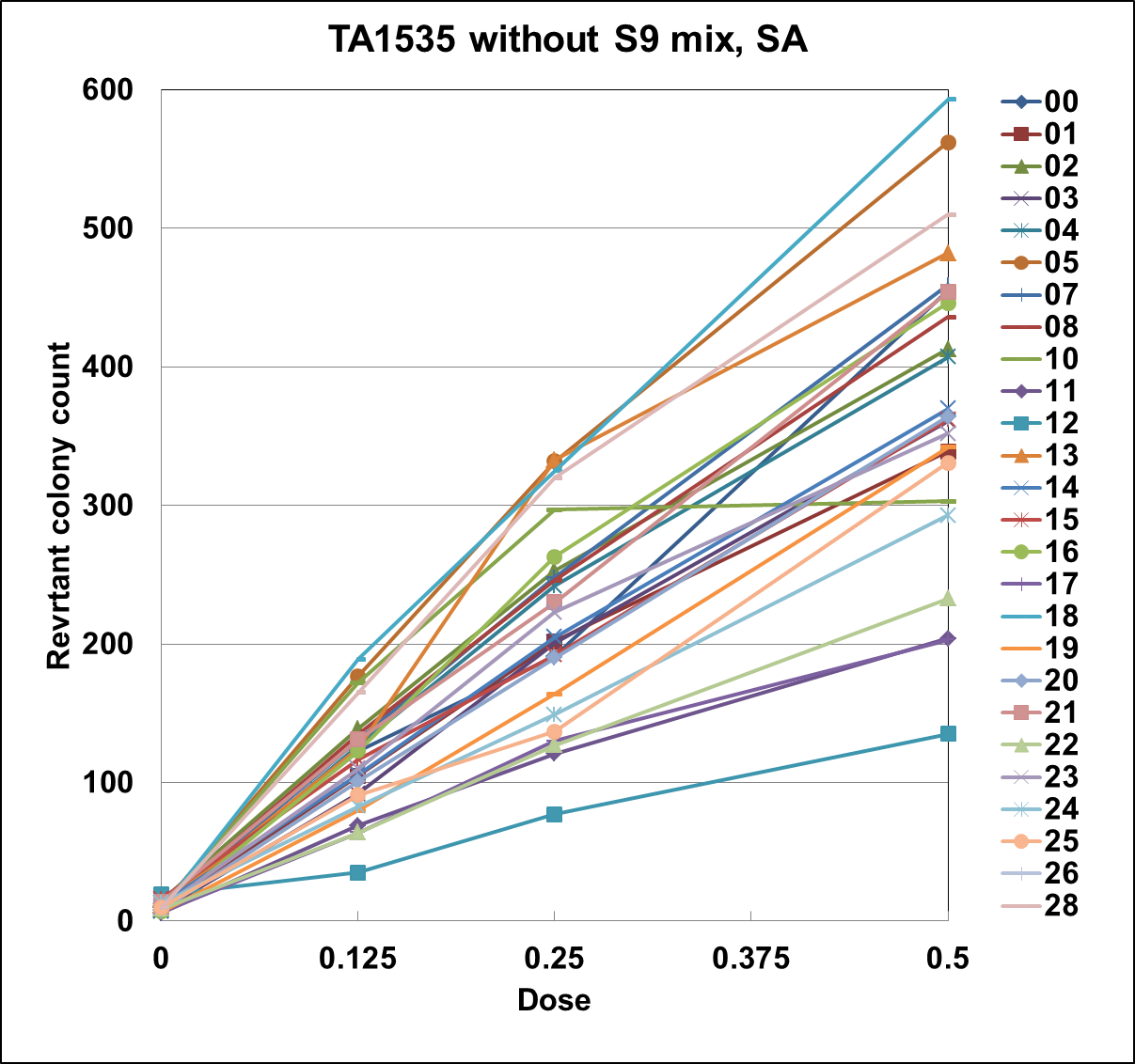

Supplement: Supplementary file 4 — Figure S3. Dose-response curves of revertant Salmonella Typhimurium strain TA1535 colonies following treatment with SA in the absence of S9 mix (a), or with 2AA in the presence of S9 mix (b). Individual dose-response curves were generated using results produced by each participating laboratory in 2016 (different colors indicate different laboratories). The doses tested were 0.125, 0.25, and 0.5 μg/plate for SA, and 0.5, 1.0, and 2.0 μg/plate for 2AA. (ODP 411 kb) [file 41021_2018_96_MOESM4_ESM.odp]

## Slide: page1
Figure S4
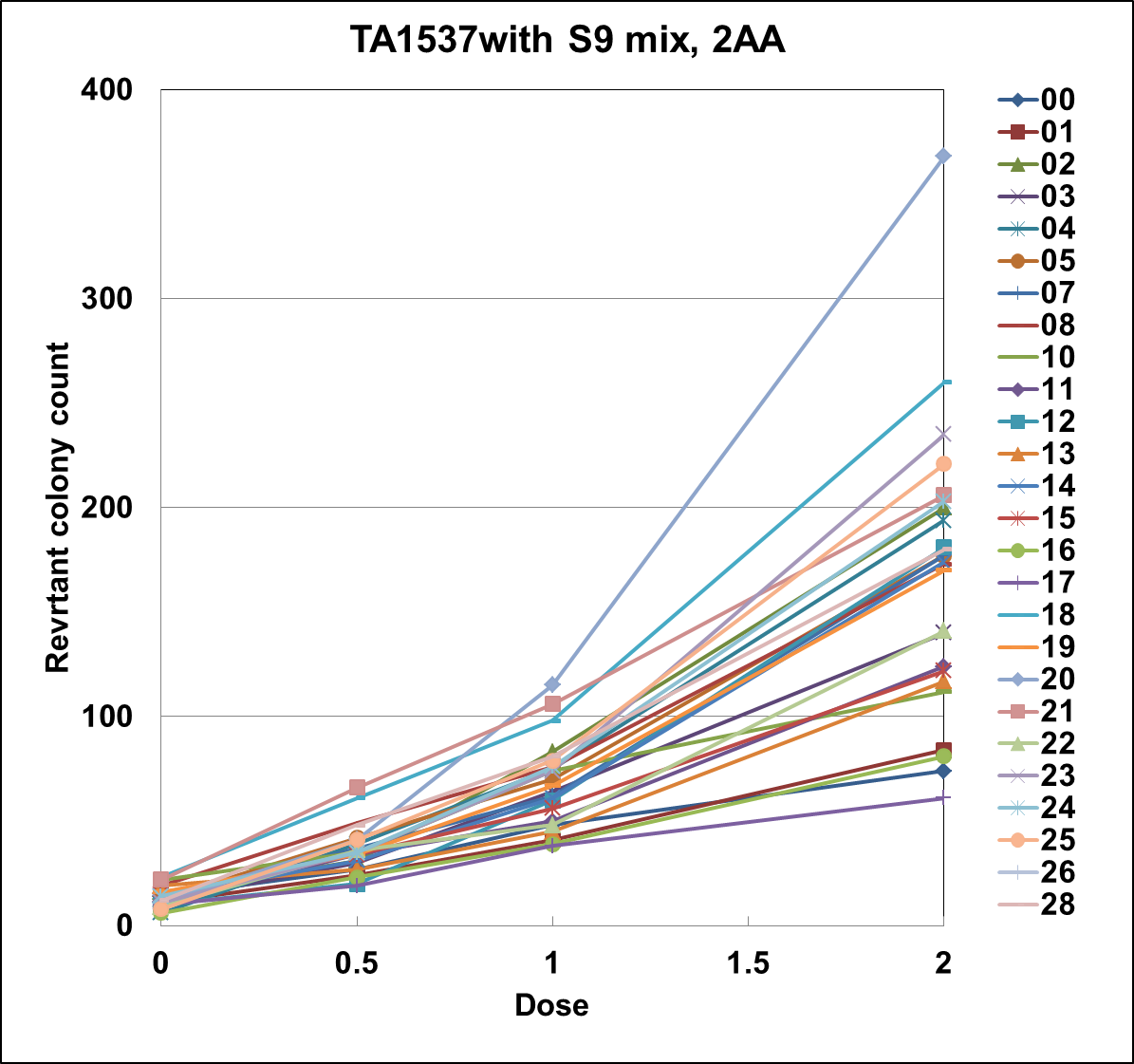

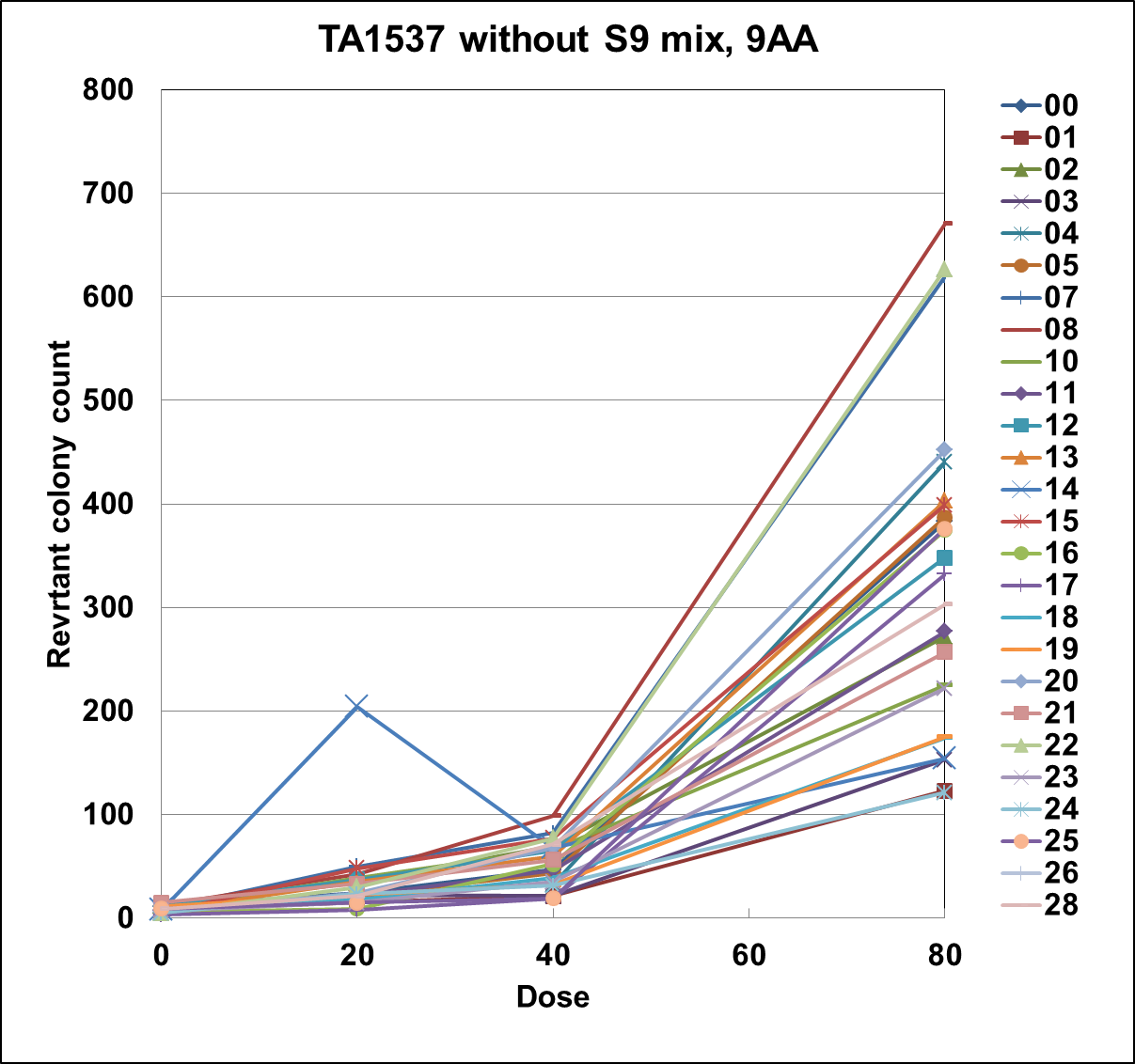

Supplement: Supplementary file 5 — Figure S4. Dose-response curves of revertant Salmonella Typhimurium strain TA1537 colonies following treatment with 9AA in the absence of S9 mix (a), or with 2AA in the presence of S9 mix (b). Individual dose-response curves were generated using results produced by each participating laboratory in 2016 (different colors indicate different laboratories). The doses tested were 20, 40, and 80 μg/plate for 9AA, and 0.5, 1.0, and 2.0 μg/plate for 2AA. (ODP 343 kb) [file 41021_2018_96_MOESM5_ESM.odp]

## Slide: page1
Figure S5
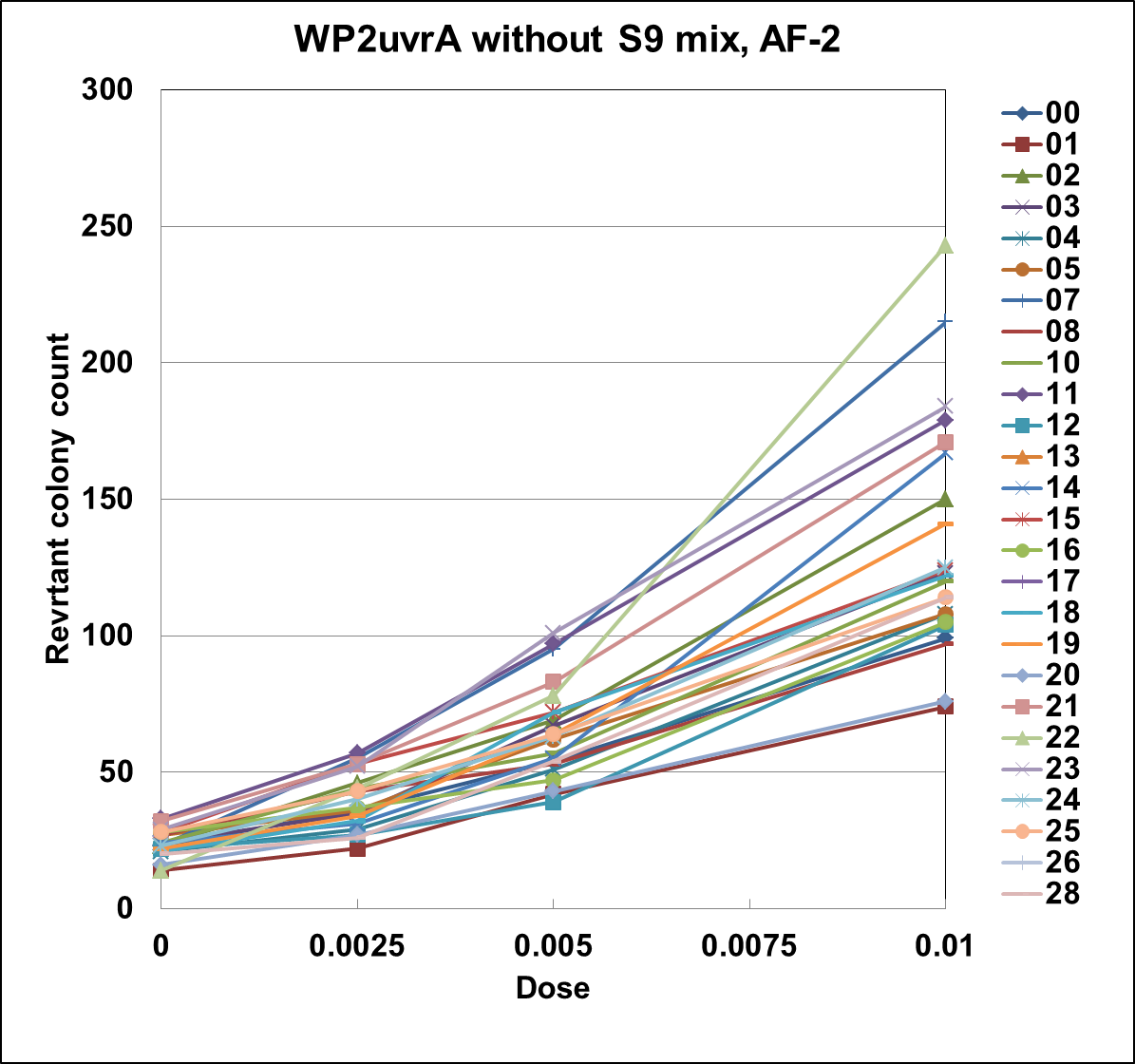

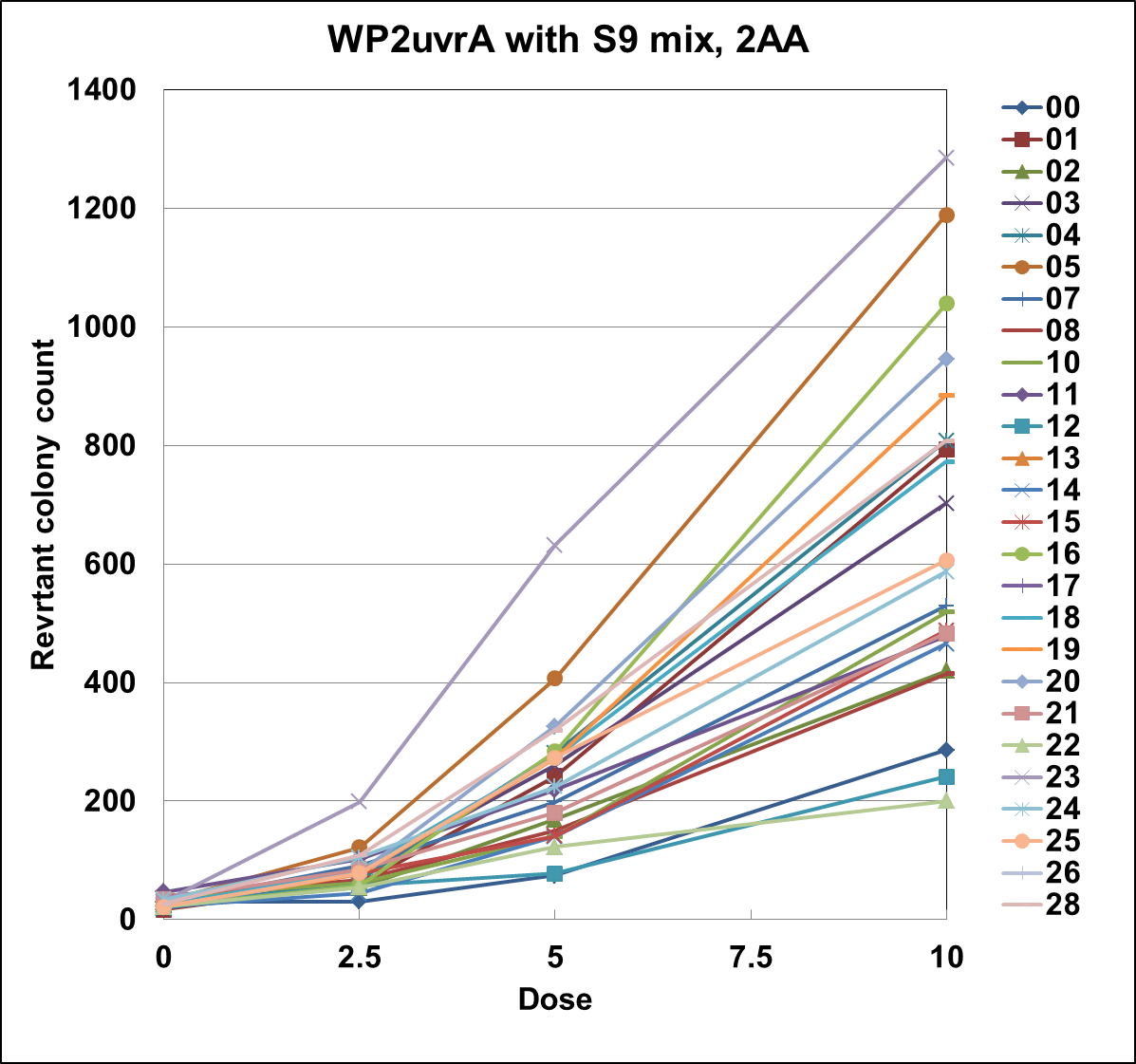

Supplement: Supplementary file 6 — Figure S5. Dose-response curves of revertant Escherichia coli strain WP2uvrA colonies following treatment with AF-2 in the absence of S9 mix (a), or with 2AA in the presence of S9 mix (b). Individual dose-response curves were generated using results produced by each participating laboratory in 2016 (different colors indicate different laboratories). The doses tested were 0.0025, 0.005, and 0.01 μg/plate for AF-2, and 2.5, 5.0, and 10 μg/plate for 2AA. (ODP 342 kb) [file 41021_2018_96_MOESM6_ESM.odp]

## Slide: page1
Figure S6
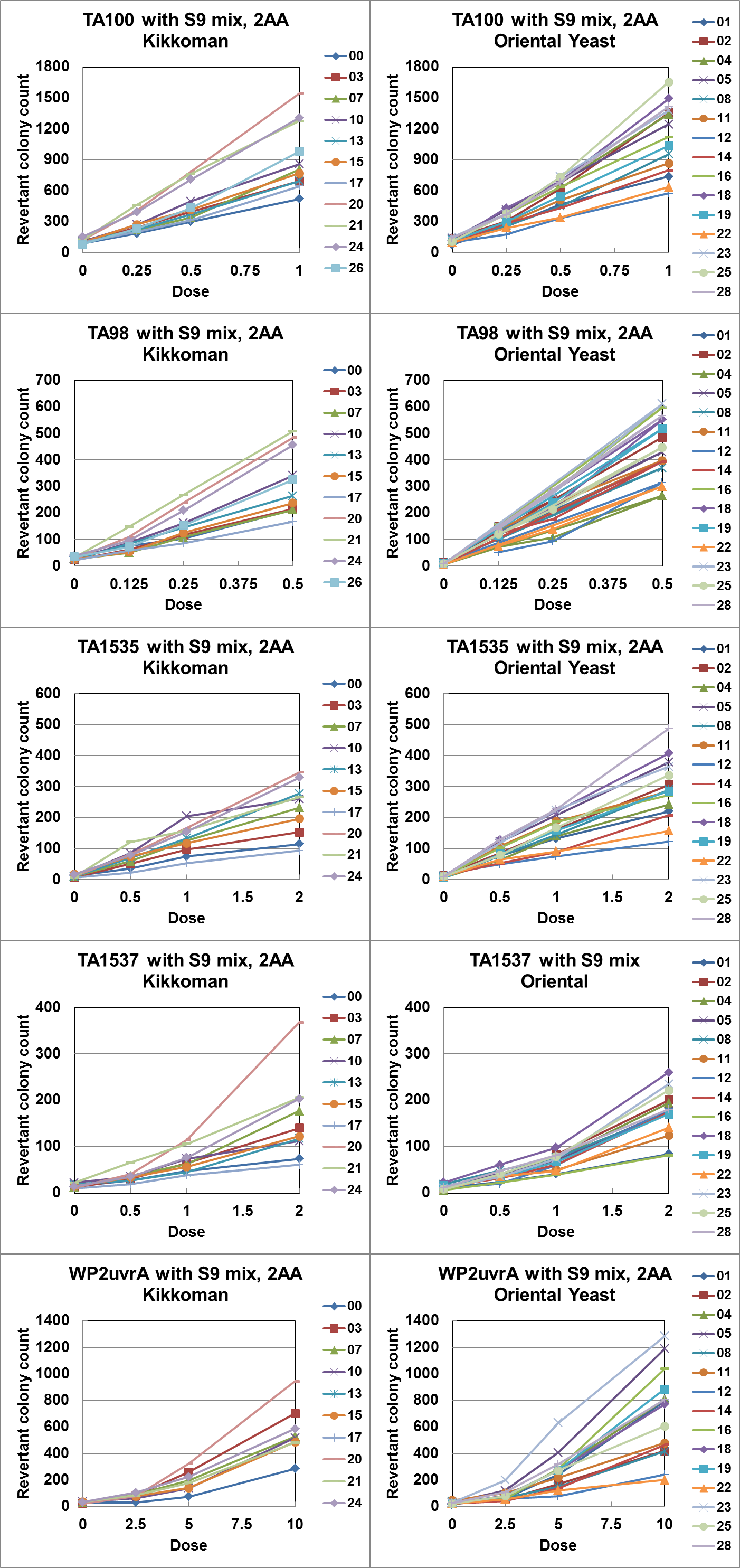

Supplement: Supplementary file 7 — Figure S6. Individual dose-response curves showing the positive control articles in the presence of S9 mix were generated from data obtained by each participating laboratory (each laboratory is indicated by a different color). The doses (in μg/plate) for each positive control article are the same as those shown in Figs. 6, 7, 8, 9, and 10. S9 fraction was purchased from Kikkoman or Oriental Yeast, and while some laboratories used that same lots, other laboratories used different lots from the same manufacturers. Laboratory identification numbers are indicated on the right-hand side of each figure. (ODP 516 kb) [file 41021_2018_96_MOESM7_ESM.odp]
